# The immunoregulatory landscape of human tuberculosis granulomas

**DOI:** 10.1038/s41590-021-01121-x

**Published:** 2022-01-20

**Authors:** Erin F. McCaffrey, Michele Donato, Leeat Keren, Zhenghao Chen, Alea Delmastro, Megan B. Fitzpatrick, Sanjana Gupta, Noah F. Greenwald, Alex Baranski, William Graf, Rashmi Kumar, Marc Bosse, Christine Camacho Fullaway, Pratista K. Ramdial, Erna Forgó, Vladimir Jojic, David Van Valen, Smriti Mehra, Shabaana A. Khader, Sean C. Bendall, Matt van de Rijn, Daniel Kalman, Deepak Kaushal, Robert L. Hunter, Niaz Banaei, Adrie J. C. Steyn, Purvesh Khatri, Michael Angelo

**Affiliations:** 1 Department of Pathology, Stanford University School of Medicine, Stanford, CA USA; 2 Department of Medicine, Division of Biomedical Informatics Research, Stanford University School of Medicine, Stanford, CA USA; 3 Institute for Immunity, Transplantation and Infection, Stanford University School of Medicine, Stanford, CA USA; 4 Department of Molecular Cell Biology, Weizmann Institute of Science, Rehovot, Israel; 5 Calico Life Sciences LLC, South San Francisco, CA USA; 6 Department of Pathology, University of Wisconsin, Madison, WI USA; 7 Division of Biology and Bioengineering, California Institute of Technology, Pasadena, CA USA; 8 Africa Health Research Institute, University of KwaZulu-Natal, Durban, South Africa; 9 Texas Biomedical Research Institute, San Antonio, TX USA; 10 Department of Molecular Microbiology, Washington University School of Medicine, St. Louis, MO USA; 11 Department of Pathology and Laboratory Medicine, Emory University School of Medicine, Atlanta, GA USA; 12 Southwest National Primate Research Center, Texas Biomedical Research Institute, San Antonio, TX USA; 13 Department of Pathology and Laboratory Medicine, University of Texas Health Sciences Center at Houston, Houston, TX USA; 14 Division of Infectious Diseases & Geographic Medicine, Department of Medicine, Stanford University, Stanford, CA USA; 15 Department of Microbiology, University of Alabama at Birmingham, Birmingham, AL USA

**Keywords:** Tuberculosis, Imaging the immune system, Infection

## Abstract

Tuberculosis (TB) in humans is characterized by formation of immune-rich granulomas in infected tissues, the architecture and composition of which are thought to affect disease outcome. However, our understanding of the spatial relationships that control human granulomas is limited. Here, we used multiplexed ion beam imaging by time of flight (MIBI-TOF) to image 37 proteins in tissues from patients with active TB. We constructed a comprehensive atlas that maps 19 cell subsets across 8 spatial microenvironments. This atlas shows an IFN-γ-depleted microenvironment enriched for TGF-β, regulatory T cells and IDO1^+^ PD-L1^+^ myeloid cells. In a further transcriptomic meta-analysis of peripheral blood from patients with TB, immunoregulatory trends mirror those identified by granuloma imaging. Notably, PD-L1 expression is associated with progression to active TB and treatment response. These data indicate that in TB granulomas, there are local spatially coordinated immunoregulatory programs with systemic manifestations that define active TB.

## Abstract (web summary)

MIBI-TOF is a mass spectrometry-based multiplexed imaging platform that has been used to map tumors. In this Resource article, MIBI-TOF is used to provide a spatial atlas of immune responses within human tuberculosis granulomas.

## Main

*Mycobacterium tuberculosis* (*Mtb*) infection accounts for nearly 1.5 million deaths each year^1^, prompting efforts to develop new host-directed therapies to treat TB disease. However, these efforts have been hindered by an incomplete understanding of how the human immune system responds to *Mtb*. Infection is initiated when bacteria are engulfed by phagocytic cells after being inhaled into the lungs^2,3^. This triggers an immune response that converges on formation of a granuloma, a dynamic and spatially organized tissue structure composed of macrophages, granulocytes, lymphocytes and fibroblasts. From the perspective of facilitating an effective host response, granulomas play contradictory roles. On one hand, consolidation of infected cells within the myeloid core limits dissemination by partitioning them away from uninvolved lung parenchyma. On the other, tolerogenic pathways upregulated within this region may limit bacterial clearance^4–6^.

Granuloma composition can be highly variable^7^. Even within a single individual, infection can result in granulomas with distinct histologic features that each progress independently over time^8^. Controlled infections in non-human primates have revealed that a single individual can possess well over ten granulomas, and the inflammatory profile, size and bacterial ecology of these lesions differ dramatically^9–11^. Thus, the trajectory of each granuloma varies across a spectrum between complete bacterial clearance to uncontrolled dissemination. This discordance suggests that local host–bacterial dynamics within the tissue microenvironment (ME) play a central role in determining granuloma fate. Along these lines, a growing number of studies find that granuloma structure and immune cell function are interconnected^12–15^.

Taken together, these findings suggest that TB progression is impacted by focal, spatially encoded regulatory mechanisms within the granuloma ME. Thus, understanding how these mechanisms promote bacterial clearance or persistence is critical for designing effective therapies. A necessary first step toward this goal is to characterize immune cell dynamics and regulatory pathways in human TB granulomas. However, many facets of TB granuloma pathology are human specific and difficult to emulate in model systems. This is compounded by the fact that, unlike other tissue pathologies, biopsy specimens are rarely obtained during a typical TB clinical workup. In this respect, TB is a member of a larger class of diseases, where the paucity of human material and lack of high-fidelity model systems have impeded development of new therapies.

With this in mind, we applied a stepwise investigative framework where limited amounts of archival tissue and publicly available transcriptome data are integrated to glean new insight into human-specific pathobiology in TB. We employed MIBI-TOF^16^ to chart granuloma composition across eight computationally defined spatial MEs, revealing features of highly localized immune modulation in granulomas, such as IDO1- and PD-L1-expressing myeloid cells, proliferative regulatory T cells (T_reg_ cells) and high levels of transforming growth factor β (TGF-β) alongside depletion of IFN-γ. We find that IDO1 seems to be specific to TB granulomas, whereas PD-L1 and sparsity of activated T cells are also found in another granulomatous condition, sarcoidosis. Lastly, in an orthogonal analysis of blood transcriptomes from patients with TB, we observe that similar immunoregulatory expression dynamics define systemic immunity during active TB.

## Results

### Structured immune cell composition in human TB granulomas

**Fig. 1:**
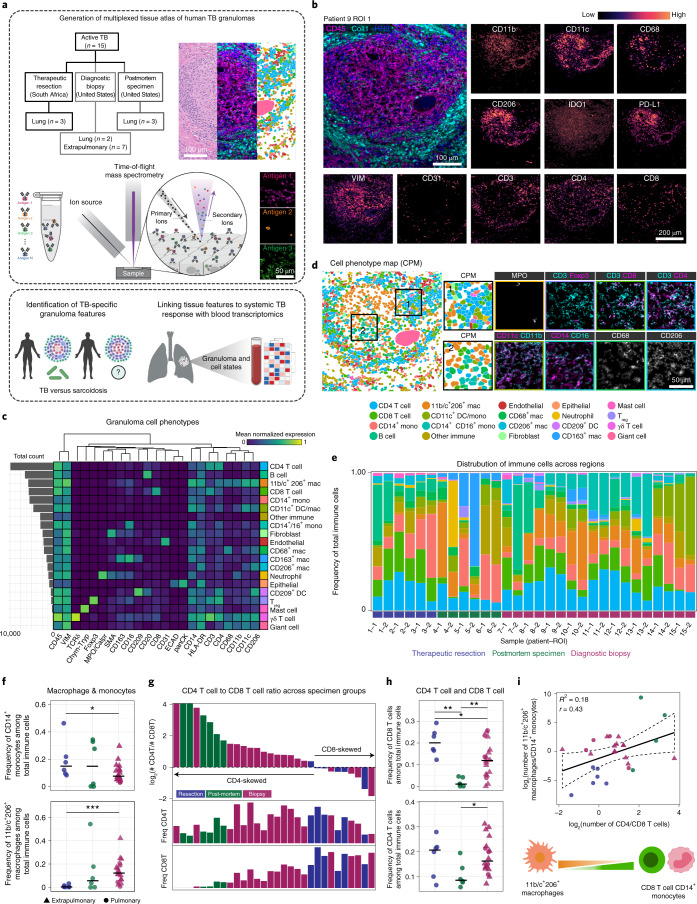
Multiplexed imaging of TB granulomas reveals structured immune cell composition. **a**, Conceptual overview of MIBI-TOF analysis of human TB granulomas, comparison with sarcoidosis and complementary analysis of systemic responses to TB. **b**, Representative images from a TB granuloma. **c**, Cell lineage assignments based on normalized expression of lineage markers (heatmap columns). Rows are ordered by absolute abundance shown on the bar plot (left), whereas columns are hierarchically clustered (Euclidean distance, average linkage). **d**, Cell identity overlaid onto the segmentation mask for a representative TB granuloma (left). Two insets (right) are shown. **e**, The relative abundance of immune cell types across all TB FOVs with cell types ordered by decreasing median abundance and bars ordered by specimen origin (resection, blue; postmortem, green; diagnostic biopsy, red). **f**, Frequency of CD14^+^ monocytes and 11b/c^+^ 206^+^ macrophages among total immune cells colored by specimen origin. Line represents the median. **g**, The CD4^+^ T cell/CD8^+^ T cell ratio represented as a log_2_ fold change for each TB FOV (top) colored by specimen origin (top) and frequency of CD4^+^ T cells (middle) and CD8^+^ T cells (bottom) among total immune cells. **h**, Frequency of CD4^+^ and CD8^+^ T cells among total immune cells colored by specimen origin. Line represents the median. **i**, Linear relationship between the CD4^+^ T cell/CD8^+^ T cell ratio and 11b/c^+^ 206^+^ macrophage/CD14^+^ monocyte ratio. Linear regression (black solid line) with 95% confidence interval (CI; black dashed line) displayed. Significance was established with a *t* test (two tailed). Unless specified, all other *P* values were calculated with a Wilcoxon rank-sum test (two tailed) (*P < 0.05; **P < 0.01; ***P < 0.001). Coll, collagen-1; mac, macrophage; mono, monocyte; MPO, myeloperoxidase; ROI, region of interest; VIM, Vimentin.

To assess granuloma composition and architecture in TB, we curated a cohort of actively infected human tissues. Archival formalin-fixed paraffin-embedded (FFPE) specimens from patients treated in the United States or South Africa were procured from Stanford Health Care and University of Texas Health Science Center or University of KwaZulu-Natal, Inkosi Albert Luthuli Central Hospital, respectively (Extended Data Table 1). The South African cohort comprised pulmonary tissues from patients undergoing therapeutic resection for advanced TB (*n* = 3), whereas a subset of US specimens came from postmortem autopsy lung tissues from patients with fatal TB (*n* = 3). Although TB disease typically manifests in the lung, infection can disseminate to extrapulmonary sites^17^. To characterize TB infection at an earlier stage and assess how granuloma composition varies with infection site, we included diagnostic biopsy specimens from lung (*n* = 2), pleural cavity (*n* = 3), lymph node (*n* = 2), vertebrae (*n* = 1) and endometrium (*n* = 1) (Fig. 1a).

**Extended Data Fig. 1:**
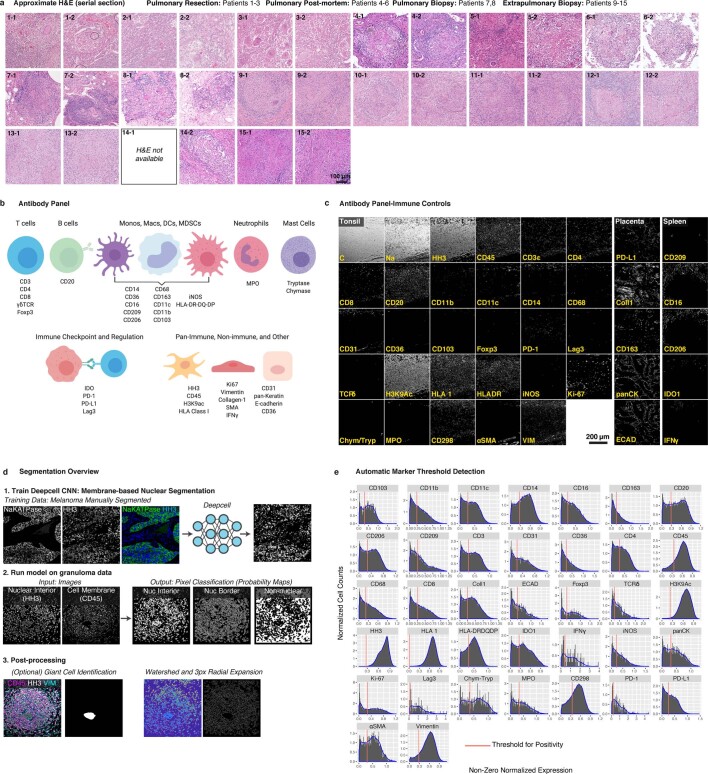
Multiplexed imaging of human TB granulomas. **a**, Hematoxylin and eosin-stained serial sections of FOVs for MIBI-TOF imaging. **b**, Multiplexed antibody panel grouped by marker category. **c**, Grayscale images of endogenous ion signal and proteins in control tissues (tonsil, spleen, placenta). **d**, Workflow for Deepcell-based segmentation of single cells from multiplexed images. **e**, Histograms of non-zero signal for all proteins from single-cell data. Blue line represents Gaussian smoothed density fit of histogram. Red line represents automatically identified threshold for marker positivity.

Each specimen was reviewed by an anatomic pathologist and screened to include the presence of active granulomatous inflammation (Extended Data Fig. 1a). MIBI-TOF was subsequently used to image two 500 μm × 500 μm fields of view (FOVs) per tissue after staining with a 37-plex panel of metal-labeled antibodies (Fig. 1b, Extended Data Fig. 1b,c and Extended Data Table 2) (ref. ^16^). The antibody panel included markers to phenotype major immune and nonimmune cell lineages, including lymphocytes, macrophages, granulocytes, stroma and epithelium. The panel also included antibodies for 12 functional markers, including those with well-documented immunoregulatory activity, such as PD-1, Lag3, PD-L1 and IDO1.

**Extended Data Fig. 2:**
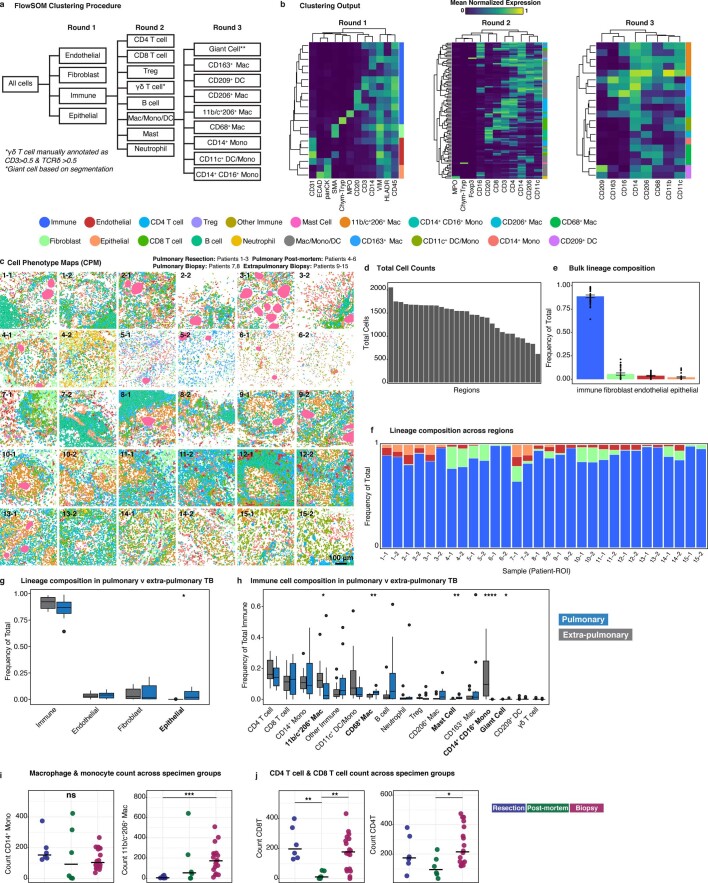
Single-cell phenotypic composition of human TB granulomas. **a**, Conceptual overview of hierarchical FlowSOM algorithm application. **b**, Heatmap of cell lineages clustered by mean normalized protein expression of markers shown along columns. **c**, Cell phenotype maps for all FOVs. **d**, Total cell counts across all FOVs sorted by descending order. **e**, Major cell lineage composition across all FOVs. Bars represent mean ± SEM (n = 30). **f**, Major cell lineage frequency of total cells broken down by FOV. **g**, Frequency of major lineages in pulmonary (blue) versus extrapulmonary (grey) TB granulomas. **h**, Frequency of immune cell subsets (of total immune cells) in pulmonary (blue) versus extrapulmonary (grey) TB granulomas. **i**, Count of CD14^+^ monocytes and 11b/c^+^ 206^+^ macrophages cells colored by specimen origin. Line represents the median. **j**, Count of CD4^+^ and CD8^+^ T cells colored by specimen origin. Line represents the median. Boxplots display the median and interquartile range (IQR, 25-75%) with whiskers representing the upper- and lower-quartile ± 1.5*IQR. P-values were determined with a Wilcoxon Rank Sum Test (two-tailed) where: * p < 0.05, ** p < 0.01, *** p < 0.001.

To extract single cells, multiplexed imaging data were processed with a low-level pipeline prior to single-cell segmentation (Fig. 1a and Extended Data Fig. 1d)^18–20^. Each FOV contained an average of ~1,410 single cells (s.d. = 343) (Extended Data Fig. 2d). FlowSOM (Extended Data Fig. 2a,b) was employed to phenotype 19 unique cell subsets (Fig. 1c) (ref. ^21^). For each image, FlowSOM clusters and segmentation masks were combined to generate cell phenotype maps (CPMs), where each cell is labeled by its phenotype (Fig. 1d and Extended Data Fig. 2c).

Granuloma composition was predominated in most lesions by T cells and myeloid cells, (average myeloid/lymphoid ratio = 2.4, s.d. = 2.4). Myelomonocytic cells comprised multiple subsets of macrophages, dendritic cells (DCs) and monocytes that were distinguished by varying degrees of coexpression of CD11c, CD11b, CD209, CD68, CD14, CD16 and CD206 (Fig. 1c,e). Granulocytes consisted of neutrophils (mean = 2.5%, s.d. = 8.8%, of total immune cells) and mast cells (0.6% ± 0.9). We also identified γδ T cells (0.1% ± 0.3), CD209^+^ DCs (0.2% ± 0.6) and T_reg_ cells (1.0% ± 1.7), highlighting the capability of our approach to enumerate low-abundance cell populations that are suggested to play a key role in granuloma pathology. In line with increased vascularization in active disease^22,23^, nonimmune cells were predominated in most lesions by endothelial cells (3.7% ± 2.8, of total cells), whereas fibroblasts (5.6% ± 6.5) and epithelial cells (2.0% ± 3.7) varied between lesions (Extended Data Fig. 2e,f). Altogether, we assigned 94% (*n* = 39,709 single cells) of cells to 19 subsets that ranged in frequency from 0.1% to 15% across our dataset.

To understand the relationship between granuloma composition and organ site, we compared cell abundances between pulmonary and extrapulmonary tissues. Interestingly, we found the vast majority of subsets (13/19) occurred in similar proportions irrespective of organ site (Extended Data Fig. 2g,h). However, pulmonary tissues displayed increased proportions of mast cells (*P* = 0.002, Wilcoxon rank sum), CD68^+^ macrophages (*P* = 0.007), and multinucleated giant cells (MNGCs; *P* = 0.03), along with a slight decrease in CD11b^+^ CD11c^+^ macrophages (*P* = 0.02). Likewise, the presence of CD14^+^ CD16^+^ intermediate monocytes was nearly exclusive to extrapulmonary tissues (*P* = 6 × 10^−5^).

Given the subtle differences across organ sites, we next evaluated how granuloma composition varied with clinical origin. For this, we compared diagnostic biopsy specimens with advanced disease in postmortem and resection tissues (Fig. 1e–h and Extended Data Fig. 2i,j). First, we found differences in CD8^+^ T cell frequency drove skewing of the CD4^+^ to CD8^+^ T cell ratio between groups, with postmortem and resection tissues exhibiting the lowest and highest proportion of CD8^+^ T cells, respectively (*P* = 0.005, Wilcoxon rank sum). Second, therapeutic resections were preferentially depleted of CD11b^+^CD11c^+^ macrophages and instead enriched for CD14^+^ monocytes. Notably, we found these two trends were moderately correlated (*R*^2^ = 0.18, *r* = 0.43 and *P* = 0.026*, t* test; Fig. 1i). Although both sample types were from patients with advanced disease, the clinical course of patients undergoing resection differed from that of postmortem specimens due to the acute, presurgical antimicrobial treatment the patient had received. Thus, it is possible that coordinated CD8^+^ T cell and monocyte recruitment is driven by presurgical antimicrobial therapy, although other differences between these specimens should be considered. Altogether, this comprehensive cell census revealed distinct types of granulomas that are defined by immune cell frequency and associate with TB disease status.

### Mapping spatially coordinated biological responses in TB granulomas

**Fig. 2:**
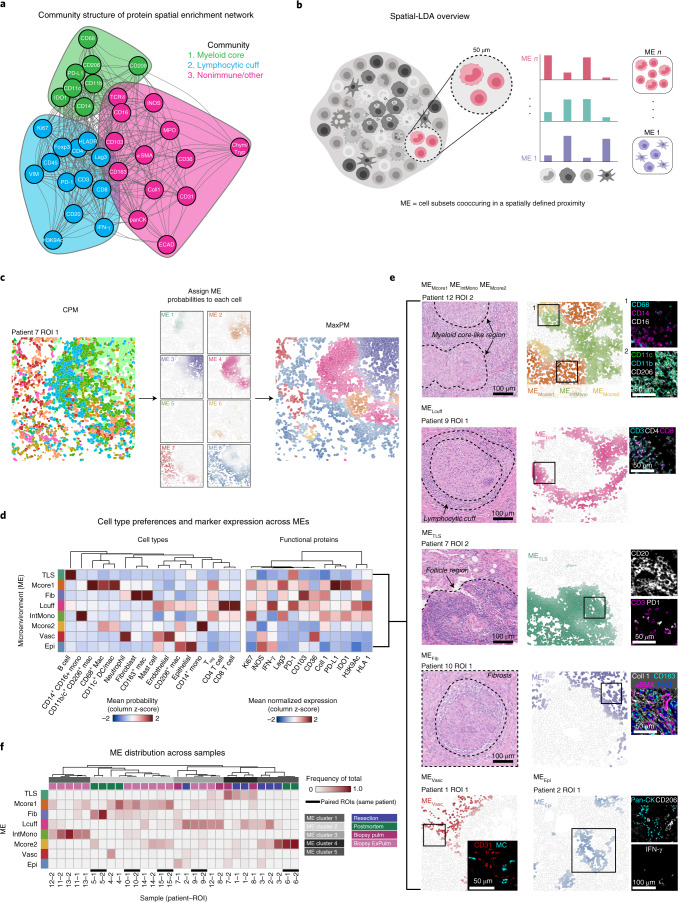
Spatial analysis of granuloma protein expression and cellular MEs. **a**, Positive spatial enrichments (average z-score >0) between protein pairs as a weighted, undirected network (edge weight is proportional to average z-score) with three communities (myeloid core, green; lymphocytic cuff, blue; nonimmune/other, pink). **b**, Conceptual overview of spatial-LDA. **c**, Cell probability map (left), max probability map (right), and ME probability for 8 MEs (middle, scaled 0 to 1) for a representative TB granuloma. **d**, Heatmap of ME preferences for all subsets (standardized mean ME loading) with hierarchical clustering (Euclidean distance, complete linkage) and mean normalized expression of functional markers (probability weighted mean) with columns hierarchically clustered (Euclidean distance, complete linkage). **e**, Biological classification of MEs. **f**, Frequency of all MEs per FOV. Heatmap columns are hierarchically clustered (Pearson correlation, complete linkage). Paired ROIs from the same patient annotated with a black bar. ME cluster and sample clinical origin annotated below dendrogram. ExPulm, extrapulmonary; MC, mast cell; pulm, pulmonary; HH3, histone H3; Pan-CK, pan-cytokeratin.

**Extended Data Fig. 3:**
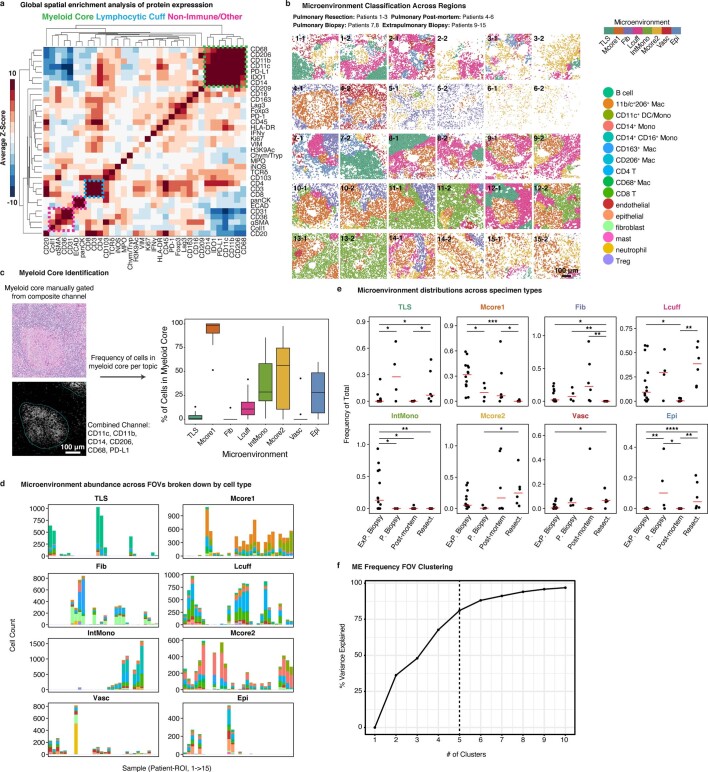
Spatial protein enrichment and microenvironment modeling of TB granulomas. **a**, Spatial enrichments of protein expression averaged across all TB granuloma FOVs and visualized as a heatmap hierarchically clustered (Euclidean distance, average linkage). Dashed boxes correspond to modules of protein enrichment corresponding to the myeloid core (green), lymphocytic cuff (blue), and a nonimmune/other niche (pink). **b**, Max probability maps (MaxPM) for all FOVs. **c**, Representative hematoxylin & eosin and combined myeloid channel of a pleural TB FOV for identification of the myeloid core (left) and frequency of cells in the myeloid core across microenvironments (right). Boxplots display the median and interquartile range (IQR, 25-75%) with whiskers representing the upper- and lower-quartile ± 1.5*IQR (n = 15). **d**, Counts of cells broken down by phenotype across all FOVs and microenvironments. **e**, Frequency of cells across microenvironments broken down by specimen type. Line represents the median (n = 30). **f**, Percent variance explained per clusters based on clustering in Fig. 2f. P-values were determined with a Wilcoxon Rank Sum Test (two-tailed) where: ns p > 0.05, * p < 0.05, ** p < 0.01, *** p < 0.001, **** p < 0.0001.

To examine how granuloma structure and function are interrelated, we conducted a spatial enrichment analysis that quantified the degree of co-occurrence between protein pairs (Extended Data Fig. 3a) (ref.^18^). Enrichment scores were used to construct an interaction network that was analyzed using a community detection algorithm^24^ (Fig. 2a). This revealed three spatial modules consistent with canonical granuloma structures, including the myeloid core, lymphocytic cuff and stromal compartment. Intriguingly, these modules also revealed granular, previously unknown features linking cell function to spatial organization, such as association of the lymphocytic cuff with H3K9Ac and the myeloid core with IDO1 and PD-L1. Notably, this linkage was present irrespective of specimen type, suggesting granuloma structure and function are coupled and conserved within these compartments (Fig. 2a).

These findings motivated us to assess how single-cell function and granuloma structure are connected. Therefore, we employed spatial latent Dirichlet allocation (spatial-LDA)^25^ to discover and assign cellular MEs to each cell, where an ME is defined by cell types spatially co-occurring across the cohort (Fig. 2b). Using this approach, we identified eight MEs for summarizing the local frequency of cell subsets within a 50-μm radius of a target cell (Fig. 2b,c). We then labeled each cell with its highest-probability ME to generate a maximum probability map (MaxPM; Fig. 2c and Extended Data Fig. 3b). Through this approach, granuloma composition and structure were summarized with two spatial representations, a CPM and MaxPM, where cells are labeled by cell type or ME, respectively (Fig. 2c).

This allowed us to annotate canonical features of granuloma histology in an unbiased fashion while revealing previously unrecognized niches (Fig. 2d,e). The majority of granuloma macrophages and monocytes belonged to one of three myeloid MEs (ME_Mcore1_, ME_IntMono_ and ME_Mcore2_). ME_Mcore1_ and ME_Mcore2_ were found to some degree across all specimen types, whereas ME_IntMono_ was notably enriched in extrapulmonary diagnostic biopsy specimens (Fig. 2f and Extended Data Figs. 3d,e and 2h). ME_Mcore1_ exhibited the strongest preference for the sharply demarcated granuloma core region (Extended Data Fig. 3c). In contrast, ME_Mcore2_ exhibited diffuse distribution of CD14^+^ monocytes and was enriched in therapeutic resections relative to pulmonary biopsy specimens, where ME_Mcore1_ was prevalent (Fig. 2d,e). In line with this, ME_Mcore1_ was highly enriched in extrapulmonary tissues and moderately to highly abundant in postmortem specimens (Extended Data Fig. 3e). Lastly, ME_IntMono_ exhibited the lowest preference for the canonical myeloid core and was enriched for CD14^+^CD16^+^ intermediate monocytes (Fig. 2d,e and Extended Data Fig. 3c).

Next, we annotated two lymphoid MEs, the lymphocytic cuff (ME_Lcuff_) and tertiary lymphoid structures (ME_TLS_). ME_Lcuff_ aligned with the second canonical granuloma ME (the lymphocytic cuff) and was composed of CD4^+^ and CD8^+^ T cells (Fig. 2d,e). ME_TLS_ was predominated by B cells, with sparse numbers of follicular helper T cells (CD4^+^ PD-1^+^), consistent with TLSs (confirmed by hematoxylin and eosin (H&E); Fig. 2e) (refs. ^26,27^). This ME was highly abundant in FOVs that were B cell enriched across specimen groups (Figs. 1e and 2f).

In our composition analysis, we observed that some granulomas exhibited a fibrotic wound-healing response with fibroblasts and CD163^+^ M2-like macrophages (Fig. 1e). Spatial-LDA revealed these cells colocalized within ME fibrosis (ME_Fib_), where CD36, a fibroblast marker, and collagen-1, a marker for fibrosis, were expressed (Fig. 2d,e)^28^. The last two MEs represented less characterized cellular environments in TB infection. ME vasculature (ME_Vasc_) was predominated by blood vessels, neutrophils and mast cells, whereas ME_Epi_ (lung parenchyma) was composed of IFN-γ^+^ epithelial cells and CD206^+^ alveolar-like macrophages (Fig. 2d,e). Given that these cells are known to participate in angiogenesis, tissue repair, and immune cell recruitment^29^, perivascular localization of mast cells in the granuloma could suggest their involvement in these processes, especially considering the association between mast cell quantity and local bacterial burden^30^. On the other hand, because ME_Vasc_ was modestly lower in extrapulmonary biopsy specimens than pulmonary biopsy specimens (*P* = 0.06, Wilcoxon rank sum) and significantly lower than pulmonary resections (*P* = 0.04) (Fig. 2f and Extended Data Fig. 3d,e), this result may reflect organ-specific differences in the association between vascularity and granulocytes or abundance of tissue resident mast cells.

Given the association between granuloma composition and clinical origin, we next sought to determine whether this relationship applied to granuloma structure. Using a correlation-based approach, we found that five ME frequency clusters accounted for 81% of variance in our dataset (Fig. 2f and Extended Data Fig. 3f). Notably, four out of five of these clusters contained samples from more than one group, supporting a recurrent spatial framework where granuloma composition and structure are coupled in a manner that is clinically agnostic (Fig. 2f). Altogether, this result suggests that MEs capture spatial features that are not discernible by bulk cell composition alone and indicate spatially coordinated biological responses.

### Granuloma myeloid cells express an immunoregulatory program

**Fig. 3:**
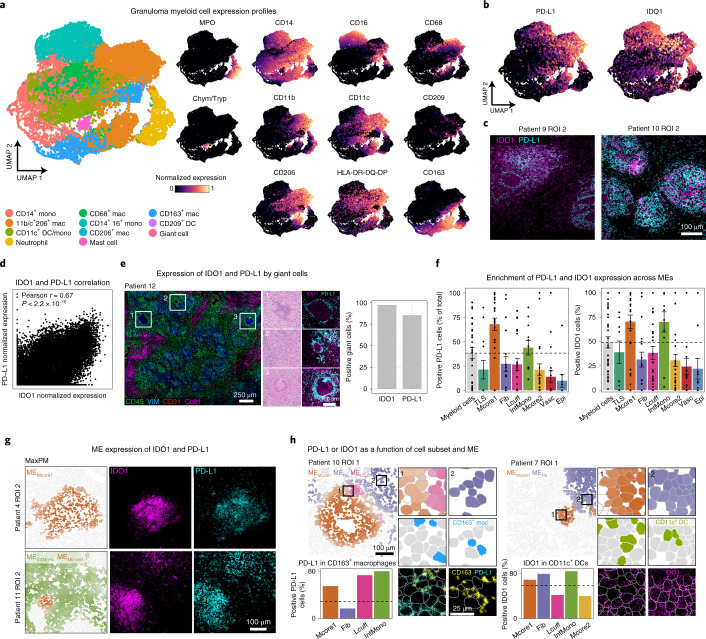
Granuloma myeloid cells express a spatially coordinated immunoregulatory program. **a**, UMAP visualization of all myeloid populations across all TB FOVs colored by subset (left) and normalized expression of phenotypic markers used to delineate subsets. **b**, IDO1 and PD-L1 normalized expression overlaid on the UMAP. **c**, Representative images of TB granulomas showing expression of IDO1 (magenta) and PD-L1 (cyan). **d**, PD-L1 and IDO1 expression values across all myeloid cells as a biaxial scatter plot. Plot displays Pearson’s *r* and *P* value calculated by a *t* test (two tailed). **e**, Giant cells identified from a MIBI-scanned TB sample (CD45, green; Vimentin, blue; CD31, red). Representative giant cells displayed in zoomed insets (lower left) with H&E staining or IDO1 (magenta) and PD-L1 (cyan) expression. Bar plot displays the percentage of IDO1^+^ and PD-L1^+^ giant cells (*n* = 34, normalized expression >0). **f**, The frequency of IDO1^+^ and PD-L1^+^ nongranulocytic myeloid cells in aggregate and broken down by ME. Bars represent mean ± s.e.m. (*n* = 30)**. g**, ME_Mcore1_ and ME_IntMono_ maximum probability maps and representative images of a pulmonary (top) and pleural (bottom) TB sample showing expression of IDO1 (magenta) and PD-L1 (cyan). **h**, Frequency of PD-L1^+^ CD163^+^ macrophages (left) across MEs with a representative MaxPM. Insets are colored by ME (top), cell type (blue, middle) and CD163 (yellow) and PD-L1 (cyan), with the segmentation boundaries overlaid (white). The frequency of IDO1^+^ CD11c^+^ DCs (right) across MEs with a representative MaxPM. Insets are colored by ME (top), cell type (green, middle) and IDO1 (magenta), with the segmentation boundaries overlaid (white). Dashed lines represent the total frequency of positive cells (PD-L1 or IDO1) for the indicated cell subset. HLA, human leukocyte antigen; HLA-DR-DQ-DP, HLA-DR/HLA-DQ/HLA-DP.

**Extended Data Fig. 4:**
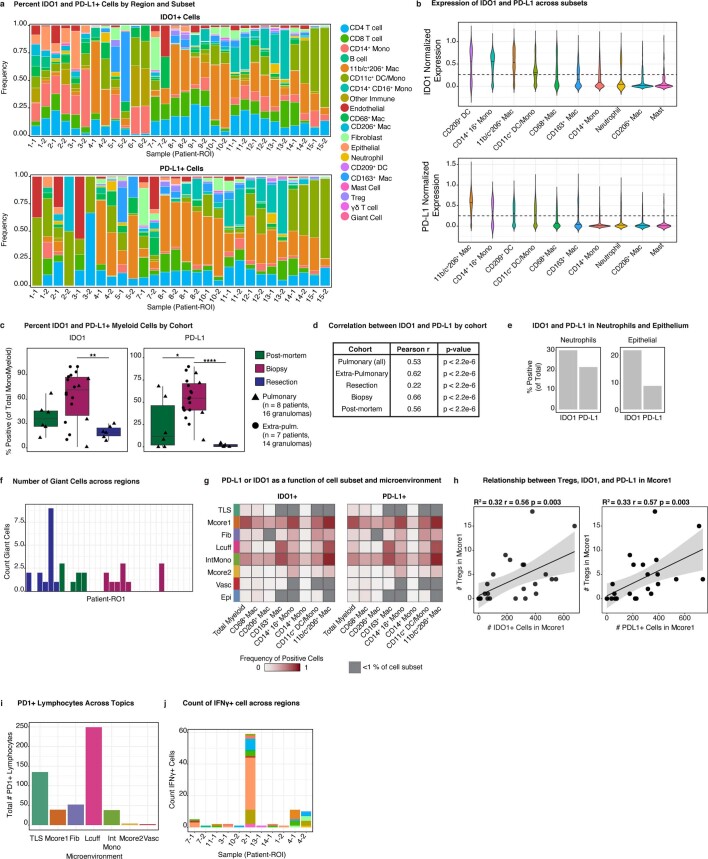
Immunoregulatory protein expression in TB granulomas. **a**, Frequency of cells positive for IDO1 (top) or PD-L1 (bottom) broken down by FOV and cell phenotype. **b**, Normalized expression of IDO1 (top) and PD-L1 (bottom) for major myeloid subsets ordered by decreasing median expression value. Dashed line indicates the cutoff for positivity for IDO1 (cutoff = 0.26) and PD-L1 (cutoff = 0.25). **c**, Frequency of IDO1^+^ or PD-L1^+^ cells (of total cells) across specimen type (postmortem specimen = green, biopsy = red, therapeutic resection = blue) with dot shape representing organ site (lung = triangle, extrapulmonary = circle). **d**, Pearson correlation coefficient and p-value determined by t-test (two-tailed) broken down by specimen type. **e**, Frequency of neutrophils (left) and epithelial cells (right) positive for PD-L1 or IDO1 across all FOVs. **f**, The count of giant cells across all regions, colored by specimen origin as in c. **g**, The frequency of IDO1^+^ and PD-L1^+^ myeloid cells for all non-granulocytic myeloid cell subsets across ME. Any ME with fewer than 1% of the total cell subset is shaded gray. **h**, Linear relationship between count of Tregs in Mcore1 with count of IDO1^+^ (left) and PD-L1^+^ (right) cells in Mcore1. Linear regression (black solid line) with 95% confidence interval (grey silhouette) displayed. Significance was established with a two-tailed t-test. **i**, Count of PD-1^+^ cells across all MEs with >0 positive cells. **j** The count of IFNγ^+^ cells in all FOVs with > 0 positive cells, colored by cell type. Unless otherwise specified all p values were determined with a Wilcoxon Rank Sum Test (two-tailed) where: * p < 0.05, ** p < 0.01, **** p < 0.0001.

Spatial modeling of granulomas revealed myeloid-rich regions of the granuloma are characterized by expression of IDO1 and PD-L1 (Fig. 2a,d). Given the tolerogenic role of these proteins^31–35^, we sought to characterize the cellular and spatial nature of immunoregulatory phenotypes in the myeloid compartment. Coexpression of PD-L1 and IDO1 was correlated (Pearson *r* = 0.67, *P* < 2.2 ×10^−16^, *t* test) across 11 granulocyte, macrophage, monocyte and DC populations (Fig. 3a,b and Extended Data Fig. 4a,b) and was highest in CD11b^+^CD11c^+^ macrophages (Fig. 3b and Extended Data Fig. 4b), a phenotype identical to that of a recently described immunosuppressive tumor-associated macrophage^36^. CD16^+^CD14^+^ intermediate monocytes exhibited a bimodal distribution in which PD-L1 and IDO1 associated with HLA-DR downregulation, which is consistent with immune evasion that disables antigen presentation to CD4^+^ T cells (Fig. 3b and Extended Data Fig. 4b) (ref. ^37^). With respect to clinical origin, PD-L1 and IDO1 expression was correlated in all specimens but only weakly correlated in resections because of PD-L1 depletion (Extended Data Fig. 4c,d). Notably, neutrophils also expressed IDO1 or PD-L1 (Extended Data Fig. 4e). Taken with previous work identifying neutrophils that secrete anti-inflammatory cytokines in TB granulomas^38^, these findings align with a regulatory effector function. Lastly, nearly 100% of MNGCs expressed IDO1, and ~85% expressed PD-L1, a feature present across specimen groups and organ site (Fig. 3e and Extended Data Fig. 4f).

To assess how PD-L1 and IDO1 expression varies with location in the granuloma, we calculated the frequency of PD-L1^+^ and IDO1^+^ nongranulocytic myeloid cells in each ME (Fig. 3f–h and Extended Data Fig. 4g). We found the majority of cells displayed preferential, ME-specific expression that was independent of subset frequency. For example, the frequency of PD-L1-expressing CD163^+^ macrophages was highest in ME_Mcore1_ (53.5%), ME_Lcuff_ (71.6%) and ME_IntMono_ (78.3%), despite this population being most prevalent in ME_Fib_ (Fig. 3h). Similarly, IDO1-expressing CD11c^+^ DCs were most enriched in ME_Fib_ (78.9%) and ME_IntMono_ (83.7%) (Fig. 3h). Altogether, PD-L1 and IDO1 coexpression defines a newly identified, spatially coordinated immunoregulatory feature of TB granulomas. Given the observational nature of this study, a functional role cannot be directly evaluated. However, these data support the possibility of highly localized, myeloid-mediated immune suppression in the granuloma.

### Granuloma lymphocytes are sparsely activated

**Fig. 4:**
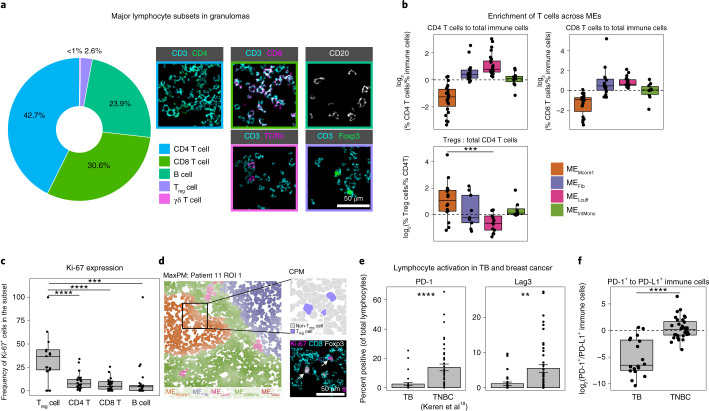
Granuloma lymphocytes display a paradoxical absence of exhaustion markers. **a**, Frequency of lymphocyte subsets in all TB FOVs pooled together (left) and representative images of each subset (right). **b**, Frequency of CD4^+^ and CD8^+^ T cells relative to the frequency of total immune cells in four MEs of interest (top). The frequency of T_reg_ cells relative to the frequency of total CD4^+^ T cells (lower left) (*n* = 30). **c**, Frequency of Ki-67^+^ cells broken down by lymphocyte subset (*n* = 30). **d**, Representative image of a TB granuloma, colored by ME assignment (left). Zoomed inset displays T_reg_ cell assignment (upper right: purple, T_reg_ cell; gray, non-T_reg_ cell) and expression of Ki-67 (magenta), CD3 (cyan) and Foxp3 (white) (lower right). **e**, Percentage of lymphocytes positive for PD-1 (left) and Lag3 (right) in all TB FOVs and TNBC. Bars represent mean ± s.e.m. (TB *n* = 30, TNBC *n* = 43). **f**, The ratio of PD-1^+^ to PD-L1^+^ immune cells represented as a log_2_ fold change in all TB FOVs and TNBC (TB *n* = 30, TNBC *n* = 43). Boxplots display the median and interquartile range (IQR; 25–75%) with whiskers representing the upper- and lower-quartile ±1.5× IQR. All *P* values were calculated with a Wilcoxon rank-sum test (two tailed) (**P < 0.01; ***P < 0.001; ****P < 0.0001).

We next wanted to evaluate to what extent the coordination between structure and function observed in myeloid cells extended to lymphocytes (Fig. 4a). On comparing the proportion of T cell subsets within each ME, T_reg_ cells (CD3^+^CD4^+^Foxp3^+^) were uniquely enriched in ME_Mcore1_ relative to the lymphocytic cuff (Fig. 4b (*P* = 0.0007, Wilcoxon rank sum)), and the total number of ME_Mcore1_-infiltrating T_reg_ cells was correlated to the number of IDO1^+^ cells (*R*^2^ = 0.32, *P* = 0.003, *t* test) and PD-L1^+^ cells (*R*^2^ = 0.33, *P* = 0.003) in this environment (Extended Data Fig. 4h). All other lymphocyte subsets were enriched in ME_Lcuff_, including Foxp3^−^CD4^+^ T cells (Fig. 4b). Furthermore, the frequency of proliferating T_reg_ cells exceeded that of all other lymphocyte subsets (Fig. 4c, *P* < 0.001, Wilcoxon rank sum). Taken together, these findings suggest that T_reg_ cells, including those actively proliferating, and immunoregulatory myeloid cells colocalize in ME_Mcore1_ to potentiate an immunomodulatory niche (Fig. 4d) (refs. ^39–42^).

Anti-inflammatory pathways can be induced as negative feedback that moderates the cytotoxic effects of unchecked immune activation^43^. In line with this, high expression of PD-L1 and IDO1 by granuloma myeloid cells would be expected to be accompanied by T cell activation in the form of checkpoint expression (e.g., PD-1 and Lag3) (ref. ^44^). For example, when examining infiltrated triple-negative breast cancer (TNBC) tumors, we found the median ratio of PD-1^+^ to PD-L1^+^ immune cells to be near unity (Fig. 4f) and the prevalence of PD-1 or Lag3 positive lymphocytes to be 13.9% and 5.5% on average, respectively (Fig. 4e).

Surprisingly, a relationship consistent with compensatory negative feedback was not observed here. We found PD-L1^+^ granuloma immune cells far outnumbered PD-1^+^ immune cells (log_2_[PD-1^+^/PD-L1^+^] = −5.1 ± 3.5; Fig. 4f). Furthermore, the small numbers of PD-1^+^ lymphocytes were largely restricted to ME_TLS_, consistent with T follicular helper cells rather than an activated phenotype (Extended Data Fig. 4i). These findings are consistent with reports from the cynomolgus macaque TB model that found low levels of PD-1, Lag3 and CTLA-4 (ref. ^45^), suggesting that IDO1 and PD-L1 expression by myeloid cells could occur independently of local signaling by activated T cells.

**Extended Data Fig. 5:**
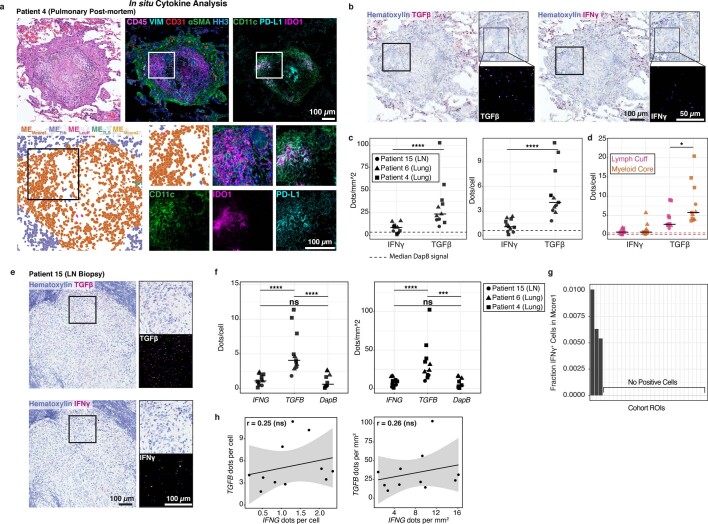
In situ hybridization of *TGFB* and *IFNG* transcripts in human TB granulomas. **a**, Representative images from a pulmonary TB granuloma section showing hematoxylin & eosin staining (upper left), MIBI-TOF images (left: CD45 = magenta, VIM = cyan, CD31 = red, αSMA = green, HH3 = blue, right: CD11c = green, PD-L1 = cyan, IDO1 = magenta) and ME assignment with zoomed insets indicated by white or black boxes. **b**, Representative chromogenic ISH of TB granuloma from a with zoomed inset indicated by black box and colored with fire LUT. **c**, Quantification of transcripts per area (left) and dots per cell (right). Solid line represents the median for probe (n = 11). Dashed line represents the median DapB signal. **d**, Quantification of transcripts per cell broken down by granuloma region (orange = myeloid core, pink = lymphocytic cuff). Solid line represents the median for probe (n = 11). Dashed line represents the median DapB signal per region type. **e**, Representative image of chromogenic ISH in a TB granuloma with zoomed insets indicated by black box. ISH signal colored with fire LUT. **f**, Quantification of TGFB, IFNG, and DapB transcripts per cell (left) and per area (right). Solid line represents the median for probe (n = 11). **g**, Proportion IFNγ^+^ cells measured by MIBI-TOF in ME_Mcore1_ as a fraction of total IFNγ^+^ cells per ROI. **h**, Linear relationship between *IFNG* and *TGFB* dots per cell (left) and dots per mm^2^ (right). Linear regression (black solid line) with 95% confidence interval (grey silhouette) displayed. Pearson correlation coefficient displayed. Significance was established with a t-test (two-tailed). Unless specified, all p values were determined with a Wilcoxon Rank Sum Test (two-tailed) where: ns p > 0.05, * p < 0.05, *** p < 0.001, **** p < 0.0001.

**Extended Data Fig. 6:**
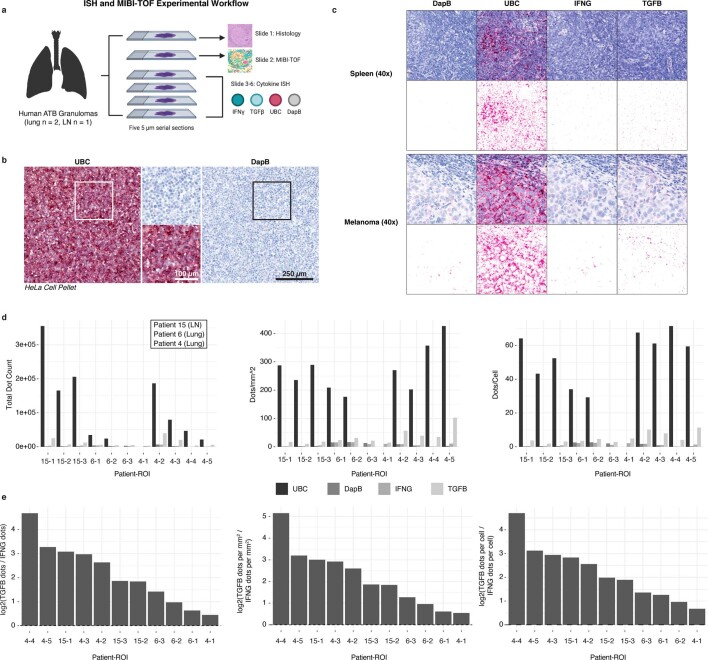
In situ hybridization workflow and supplemental analysis. **a**, Conceptual overview of combined ISH and MIBI-TOF experimental workflow. **b**, Representative images of control probes UBC ( + ) and DapB (-) in HeLa cell pellets. **c**, Representative images of all probes in human spleen and melanoma at 40x magnification. **d**, Grouped bar plots of total counts (left), area-normalized counts (middle), and cell-normalized counts (right) for all granuloma regions analyzed. **e**, The ratio of TGFB: IFNG transcripts as a log2 fold-change for total counts (left), area-normalized counts (middle), and cell-normalized counts (right) for all granuloma regions analyzed.

Considering recent work demonstrating TGF-β signaling in granulomas^46^, the combination of tolerogenic myeloid cells and T_reg_ cells along with the absence of T cell activation could indicate an immunoregulatory niche promoted through TGF-β. To measure the cytokine landscape underlying this ME, we performed in situ hybridization (ISH) for TGF-β and IFN-γ transcripts in a subset (*n* = 3) of samples (Extended Data Figs. 5 and 6). Consistent with measurements by MIBI-TOF, granulomas were depleted of IFN-γ and produced large quantities of TGF-β (Extended Data Figs. 4j and 5a–g). The majority of TGF-β was produced by cells in the myeloid core, corresponding with expression of IDO1 and PD-L1 (Extended Data Fig. 5d). However, TGF-β was also produced by cells in the lymphocytic cuff of granulomas (Extended Data Fig. 5d). There was no correlation between TGF-β and IFN-γ transcripts, suggesting that TGF-β expression may occur in the absence of T helper type 1 signaling (Extended Data Fig. 5h). These results demonstrate that TGF-β expression underlies a granuloma ME producing IDO1 and PD-L1 and promoting T_reg_ cell activity. Such an environment may potentiate a niche within the granuloma that impairs T cell activation and promotes T_reg_ cell proliferation.

### Common and diverging immunoregulatory features in TB and sarcoidosis

**Fig. 5:**
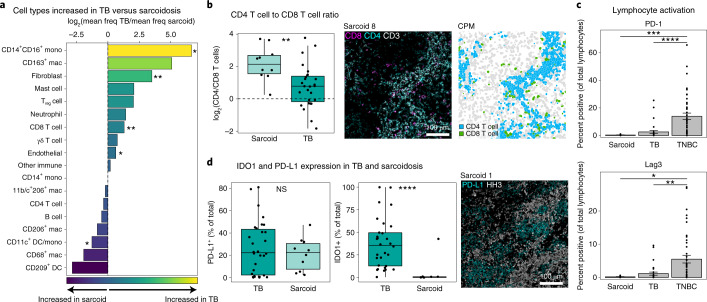
Common and diverging features of immune regulation in TB and sarcoidosis. **a**, Fold change of mean frequency of cell subsets (of total cells) in TB versus sarcoidosis with significant differences indicated with an asterisk. **b**, Comparison of the CD4^+^ T to CD8^+^ T cell ratio in TB versus sarcoidosis (TB *n* = 30, sarcoid *n* = 10). Representative image of an axillary sarcoidosis FOV showing expression of CD8 (magenta), CD4 (cyan) and CD3 (white) (left) and colored by cell type (right: blue, CD4^+^ T cell; green, CD8^+^ T cell). **c**, Percentage of lymphocytes positive for PD-1 (top) and Lag3 (bottom) in all sarcoidosis FOVs, TB FOVs and TNBC. Bars represent mean ± s.e.m. (sarcoid *n* = 10, TB *n* = 30, TNBC *n* = 43). **d**, Percentage of total cells positive for IDO1 or PD-L1 in TB and sarcoidosis (TB *n* = 30, sarcoid *n* = 10). Representative image of a sarcoidosis FOV showing expression of PD-L1 (cyan) and HH3 (white). Boxplots display the median and IQR (25–75%), with whiskers representing the upper- and lower-quartile ±1.5× IQR. All *P* values were calculated with a Wilcoxon rank-sum test (two tailed) (NS, *P* > 0.05; *P < 0.05; **P < 0.01; ***P < 0.001; ****P < 0.0001).

**Extended Data Fig. 7:**
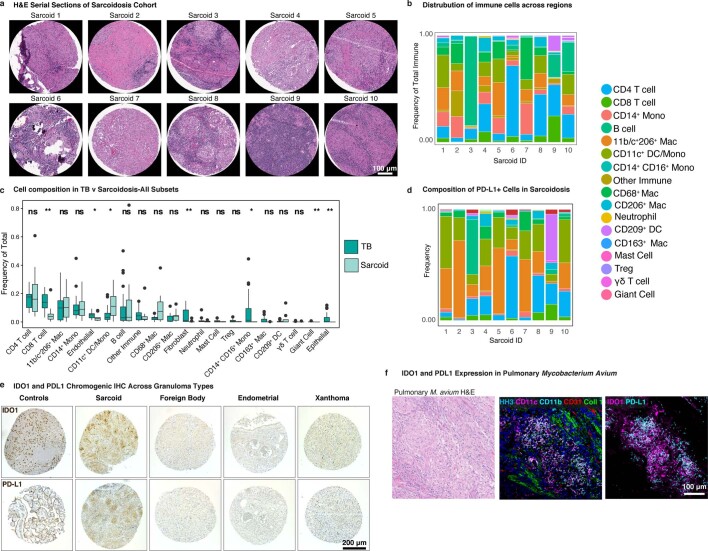
Immunoregulatory protein expression in nontuberculous granulomas. **a**, Hematoxylin & eosin-stained sections of sarcoidosis granuloma FOVs. **b**, Frequency of immune cell subsets out of total immune cells broken down by sarcoidosis FOV. **c**, Comparison of cell type frequency (out of total cells) between tuberculosis (dark green) and sarcoidosis (light green). Boxplots display the median and interquartile range (IQR, 25-75%) with whiskers representing the upper- and lower-quartile ± 1.5*IQR (TB n = 30, sarcoid n = 10). P-values were determined with a Wilcoxon Rank Sum Test (two-tailed) where: ns p > 0.05, * p < 0.05, and ** p < 0.01. **d**, Frequency of PD-L1^+^ cells across all sarcoidosis FOVs broken down by cell subset. **e**, Representative immunohistochemistry images of PD-L1 or IDO1 (brown) of controls (top = spleen, bottom=placenta), a sarcoid granuloma, xanthoma granuloma, foreign body lesion, and endometrial lesion with hematoxylin nuclear counterstaining (purple). **f**, Hematoxylin and eosin (left) and MIBI-TOF staining for major cell lineage markers (middle) or IDO1 (magenta) and PD-L1 (cyan) (right) of a representative pulmonary *Mycobacterium avium* FOV.

In addition to being the histological hallmark of TB, granulomas occur in response to foreign bodies and in autoimmune disorders, such as sarcoidosis^47^. Interestingly, gene expression studies that attempted to develop blood-based biomarkers for *Mtb* infection have struggled to differentiate TB from sarcoidosis^48,49^. To determine the extent to which features identified here overlap with other granulomatous diseases, we compared TB to ten sarcoidosis cases (Extended Data Fig. 7a). TB lesions were more variable in composition (Extended Data Fig. 7b,c) and had significantly higher frequencies of CD8^+^ T cells, fibroblasts, intermediate monocytes and giant cells and increased vascularity (Fig. 5a and Extended Data Fig. 7c). Sarcoid granulomas were heavily CD4^+^ T cell skewed, even relative to CD4-skewed TB granulomas, consistent with reports of sarcoidosis pathology being driven primarily by T helper type 17 and type 1 T cells (Fig. 5b) (refs. ^50,51^).

Like TB, sarcoid lesions were PD-1 and Lag3 depleted (Fig. 5c), despite high levels of PD-L1^+^ myeloid cells (Fig. 5d and Extended Data Fig. 7d). However, unlike TB, IDO1 expression in sarcoid samples was almost entirely absent (Fig. 5d). Because we used a conservative threshold for IDO1 and PD-L1 positivity, our analysis biased toward the moderately to strongly expressing cells present in TB granulomas and control tissues. Therefore, to evaluate the specificity of macrophage PD-L1 and IDO1 expression in *Mtb* infection, we used immunohistochemistry (IHC) to compare both proteins on a tissue microarray of granulomas from sarcoidosis (*n* = 9), foreign body uptake (*n* = 4), endometriosis (*n* = 4) and xanthomatosis (*n* = 3) (Extended Data Fig. 7e). We identified weak expression of IDO1 in several sarcoidosis lesions along with bright expression of PD-L1, as observed by MIBI-TOF (Extended Data Fig. 7e). However, IDO1^+^ and PD-L1^+^ cells were nearly absent in all xanthomas and endometrial lesions and rare in foreign body granulomas. Notably, we observed high levels of IDO1 and PD-L1 in a pulmonary *Mycobacterium avium* granuloma (Extended Data Fig. 7f). This suggests that whereas PD-L1 expression could be a broader feature of granulomatous conditions, strong coexpression of IDO1 and PD-L1 appears specific to mycobacterial granulomas.

### Immunoregulatory features are reflected across granulomas and blood

The presence of immunoregulatory features observed in our MIBI-TOF study has important implications for understanding the immunologic basis of TB disease. We next wanted to analyze these features in blood from TB patients to understand how immunoregulatory properties of granulomas are reflected during systemic immunity. Moreover, by leveraging analysis of blood, we sought to look across infection stages and correlate immune features with disease severity and progression. Therefore, we used MetaIntegrator to perform multicohort analyses using publicly available peripheral blood transcriptome profiles from healthy subjects and patients with latent or active TB infection^52,53^.

**Fig. 6:**
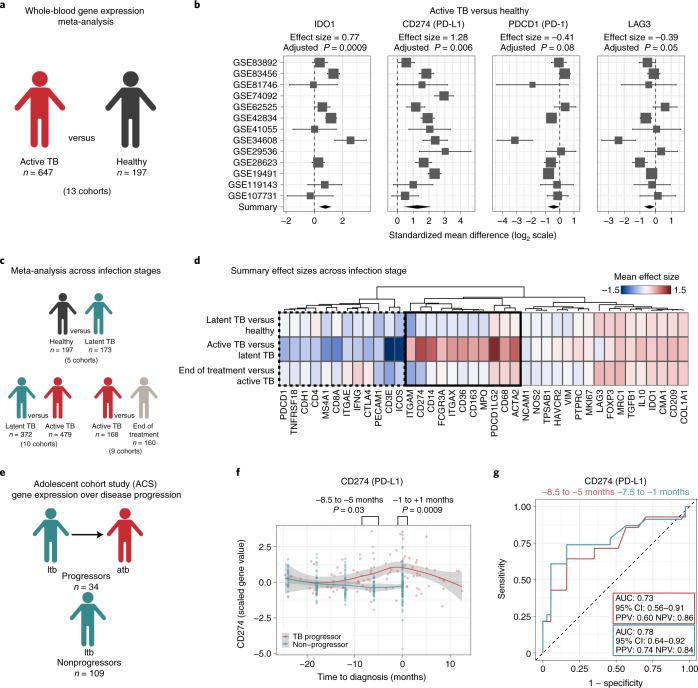
Immunoregulatory features of granulomas are reflected in the peripheral blood of TB patients. **a**, Conceptual overview of the meta-analysis of patients with active TB (*n* = 647) versus healthy controls (*n* = 197). **b**, Forest plots of gene expression differences in active TB versus healthy individuals. Cohort identifiers are shown on the *y* axis. Boxes represent the standardized mean difference in gene expression (effect size). The size of the box is proportional to the sample size of that cohort. Whiskers represent the 95% CI, and diamonds (black) represent the overall difference in gene expression between two groups by integrating the standardized mean differences across all cohorts. The width of the diamond corresponds to its 95% CI. The adjusted *P* values (*q* values, false discovery rate = 5%) for the summary effect sizes are shown above each plot. **c**, Conceptual overview of gene expression analysis across clinical infection stage. **d**, Heatmap of summary gene expression (mean effect size) values in latent TB (*n* = 173) versus healthy controls (*n* = 197), latent TB (n = 372) versus active TB (*n* = 479) and active TB (*n* = 168) versus end of treatment (*n* = 160). Clinical stage is displayed on rows, and genes are displayed across columns hierarchically clustered (Euclidean distance, complete linkage). Genes upregulated in active TB versus latent TB are shown in the solid black box, whereas downregulated genes are in the dashed black box. **e**, Conceptual overview of the ACS. **f**, PD-L1 gene expression in the ACS cohort across time prior to and after diagnosis of active TB stratified by progressors (red, *n* = 34) and nonprogressors (blue, *n* = 109). Gray silhouette represents the 95% CI of the local polynomial regression (red and blue lines). *P* values were calculated with a Welch two-sample *t* test. **g**, Receiver operating characteristic (ROC) curves of the predictive power of *CD274* (PD-L1) expression as a predictor of TB progression. atb, active TB; ltb, latent TB; NPV, negative predictive value; PPV, positive predictive value.

We first asked whether immunoregulatory signals identified in granulomas could be detected in blood by comparing gene expression data of patients with active TB (*n* = 647) to healthy controls (*n* = 197) from 13 independent cohorts (Fig. 6a). We found significant and consistent upregulation of *IDO1* and *CD274* (PD-L1) (effect size = 0.77 and 1.28, *q* = 0.0009 and 0.006 (false discovery rate = 5%), respectively) (Fig. 6b). Additionally, checkpoint depletion in lymphoid cells was corroborated, with no observed increase in expression of *PDCD1* (PD-1) or *LAG3* (effect size = −0.41 and −0.39, *q* = 0.09 and 0.05, respectively).

**Extended Data Fig. 8:**
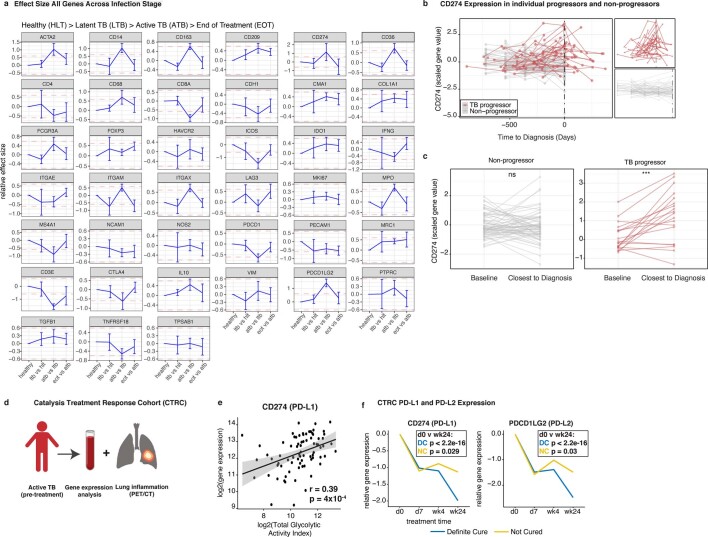
Transcriptomic analysis of peripheral blood in TB patients. **a**, Gene effect sizes in latent TB (n = 173) versus healthy controls (n = 197), latent TB (n = 372) versus active TB (n = 479), and active TB (n = 168) versus end-of-treatment (n = 160). Bars represent the mean and standard deviation. Dashed red lines represent a relative effect size of 0.6. **b**, *CD274* scaled gene expression in progressors (red) and non-progressors (gray) with zoomed insets displaying groups individually. **c**, *CD274* expression in progressors at the earliest recorded time point prior to progression and the closest time point to diagnosis with ATB (within 30 days). P-values were calculated with a one-sided paired sample t-test. **d**, Conceptual overview of the Catalysis Treatment Response Cohort (CTRC). **e**, Correlation between PD-L1 gene expression and total glycolytic activity index (TGAI) represented as log2-transformed values. Linear regression (black line) with 95% confidence interval (grey) displayed. A Pearson correlation of 0.39 (p = 4 ×10^-4^) is displayed below the linear fit. Significance was established with a t-test (two-tailed). **f** PD-L1 (left) and PD-L2 (right) gene expression across treatment time broken down by cure status (blue = definite cure and yellow = no cure). Line represents mean expression in each time point, connected across time points. P-value determined with Student’s t-test for PD-L1 expression at d0 versus wk24 in the definite cure (DC, n = 71) and not-cured (NC, n = 7) groups.

Next, we analyzed transcriptomic data from 1,549 patients across 24 cohorts to evaluate if these features were specific to active TB (Fig. 6c). In line with our MIBI-TOF analysis, differential expression of genes associated with regulatory myeloid cells (e.g., PD-L1, PD-L2, CD11b, CD11c and CD163) or T cell immune checkpoint (e.g., PD-1 and CTLA4) delineated active from latent infections (Fig. 6d). Moreover, the majority of these genes returned to baseline levels of healthy controls after antimicrobial therapy (Fig. 6d and Extended Data Fig. 8a). Taken together, these results are consistent with a shift toward myeloid-mediated immune regulation that is specific to active TB.

Because PD-L1 gene expression exhibited the largest effect size relative to healthy controls and was upregulated in granulomas, we chose to further understand its relationship with infection dynamics. First, we analyzed the adolescent cohort study (ACS) to determine if PD-L1 expression preceded progression to active disease. Latently infected individuals enrolled in this study underwent regular blood collection and were monitored for symptoms of active infection (Fig. 6e)^54,55^. Within 8.5 to 5 months of clinical diagnosis, PD-L1 transcript levels were significantly elevated in progressors and predictive of progression from latent to active disease (area under the curve = 0.73, CI 0.56-0.91) (Fig. 6f,g and Extended Data Fig. 8b,c). Strikingly, the predictive performance of PD-L1 in patients 7.5 to 1 months before progression (area under the curve = 0.78, CI 0.64-0.92) was comparable with previously published multigene signatures, despite being a single gene identified by tissue analysis^56^. Taken together, these results support that a shift toward myeloid immune regulation defines the symptomatic stage of TB infection.

We corroborated these results by analyzing the catalysis treatment response cohort to determine if PD-L1 was associated with disease burden and infection clearance (Extended Data Fig. 8d). Patients in this study provided venous blood and underwent PET/CT imaging upon TB diagnosis^57,58^. PD-L1 expression at diagnosis was directly correlated with total glycolytic activity index, a radiographic metric for lung inflammation (Extended Data Fig. 8e; Pearson *r* = 0.39, *P* = 4 × 10^−4^, *t* test). Twenty-four weeks after treatment, patients were clinically stratified as definitely cured or not cured. Relative to diagnosis, the reduction in PD-L1 expression in patients was two times greater on average in patients who were definitely cured (*n* = 71) than in patients who were not cured (*n* = 7; Extended Data Fig. 8f). A nearly identical trend was observed for PD-L2 (*PDCDLG2*; Extended Data Fig. 8f). In summary, orthogonal analysis of whole blood during TB infection demonstrated synchrony in the local and systemic immune responses during active TB disease.

## Discussion

After nearly 140 years of research into the pathophysiology of human TB, central questions remain unresolved, in part because granuloma formation and progression are difficult to emulate in tractable animal models. Considering this, we developed a framework in which a limited amount of archival tissue and publicly available transcriptomic data were used to identify features of active TB disease in humans. Using clinical specimens from three medical centers, we constructed a spatial cell atlas to relate granuloma structure and composition. We identified 19 cell subsets that organize into eight cellular MEs. TB granulomas appear to follow a consistent structural outline of spatially coordinated PD-L1 and IDO1 and myeloid core-infiltrating T_reg_ cells and a striking absence of T cell activation. Although several of these immune features have been previously identified individually^34,45,59,60^, this study demonstrates an association between myeloid cell regulatory features and reduced lymphocyte activation with spatial and single-cell resolution. Certain features, such as expression of PD-L1 and immunoregulatory macrophages, were present in noninfectious granulomas as well, pointing to universal immune programs associated with granulomatous inflammation. However, compared to other granulomatous conditions, spatially coordinated coexpression of IDO1 and PD-L1 was unique to mycobacterial granulomas.

Granulomas can display a range of disparate outcomes with respect to bacterial burden and inflammatory trajectory^10,11^. The variation in our imaging dataset suggests local outcomes may be driven by unique cellular infiltrate and structure within each granuloma. We observed that features, such as high frequency of CD8^+^ T cells, corresponded with reduced levels of more differentiated macrophage phenotypes, a profile present in therapeutic resections where PD-L1 and IDO1 expression was diminished. Because CD8^+^ T cells are important contributors to TB immunity^61,62^, understanding the immunological environments that promote their activity could reveal novel insights into immune features critical for bacterial clearance.

The high levels of PD-L1 and IDO1 observed in the near absence of PD-1 offers clues into how an immunoregulatory niche during infection is initiated and maintained. We observed that IDO1 and PD-L1 in myeloid cells were colocalized with TGFβ, further confirming the immunoregulatory nature of granuloma macrophages. This is consistent with a TGF-β- or IL-10-driven process in which production of these cytokines suppresses inflammation and induces peripheral T_reg_ cell activity^28,63,64^. These findings are supported in murine TB, where focal secretion of TGF-β within the myeloid core preferentially suppresses neighboring T cells, and in non-human primates, where granuloma formation associates with IL-10 secretion^46,65^. The next step will be to establish a causal role for TGF-β in driving immune suppression in granulomas across the full spectrum of TB.

We next assessed the extent to which the immunoregulatory features identified in archival tissue manifested in peripheral blood. In a multicohort meta-analysis, we identified signatures in blood and analyzed them with respect to disease burden and clinical outcome. As in granulomas, immunoregulatory genes such as PD-L1, IDO1 and CD163 were upregulated in blood. Similarly, genes associated with T cell activation were downregulated, consistent with the rare incidence of PD-1 or Lag3 in tissue. Importantly, the magnitude of these trends was higher in patients with active disease relative to those with latent or treated infections. PD-L1 displayed the highest effect size of all genes analyzed here. Notably, its expression correlated with pulmonary disease burden and preceded progression to active TB, in line with previous work demonstrating upregulation of *CD274* in patients with active TB^66^. It should be noted that given the low prevalence of monocytes in the blood and prior reports of upregulation of PD-L1 by neutrophils during active TB, it is likely granulocytes drive this systemic gene signature^66,67^.

Both IDO1 and PD-L1 dampen antitumor immune responses in cancer, prompting immunotherapy development^68^. Our findings suggest that similar approaches could be used to block PD-L1-mediated immune suppression in TB. However, evidence of T cell activation or exhaustion is absent in our dataset and other datasets. Given this, efficacy of PD-L1 blockade could differ substantially from PD-1 blockade. Recent reports of TB reactivation following PD-1 blockade yet fewer instances following PD-L1 blockade illustrate the paradoxical effects that occur with host-directed therapies^69–73^. In our dataset, the small numbers of PD-1 lymphocytes present were largely localized to neighboring TLSs. This raises the possibility that PD-1 blockade exacerbates immunopathology by stimulating TLS-resident and peripheral T cells while failing to activate granuloma T cells. This is supported by work in the ultra-low-dose murine model of TB, where a higher proportion of IFN-γ^+^-producing, activated CD4^+^ T cells were found at distal lung sites relative to the granuloma^46^. These data emphasize the need to map the temporal and spatial dynamics of these pathways. In line with this, a critical next step will be to connect these features to bacterial burden, inflammatory dynamics, intraindividual variability and granuloma age in a primate model that accurately recapitulates human TB pathology.

In conclusion, with our generalizable framework, we identified how cellular composition and immunoregulatory pathways in TB granulomas relate to peripheral immune responses. This has implications for developing host-directed immunotherapies and understanding the immunologic basis of failed immunity in TB. Expression of proteins such as IDO1 and PD-L1 aligns with immune-evasion mechanisms observed in the tumor-immune ME. The interface of granuloma and tumor immunobiology offers new opportunities to explore how tactics of immune evasion in tumors contribute to bacterial persistence in granulomas. Future multiplexed imaging studies of granulomas from controlled TB exposures will offer insight into how these local regulatory dynamics influence granuloma fate and, ultimately, infection outcome.

## Methods

### Human TB granuloma cohort

All human samples were acquired in accordance with institutional review board protocol 46586 (‘Generation of an Immune Atlas of Human Tuberculosis Granulomas with Multiplexed Ion Beam Imaging’). Patient consent was not acquired, as only archival clinical specimens were analyzed, with no active participation of humans. FFPE *Mtb*-infected tissues were acquired from Stanford Health Care’s tissue repository from nine patients undergoing a pretreatment diagnostic biopsy (*n* = 9). Tissues were screened to include those that were positive for acid-fast *Bacillus* and *Mtb* DNA by polymerase chain reaction. Archival surgical resection tissues were acquired from University of KwaZulu-Natal, Inkosi Albert Luthuli Central Hospital from three patients with *Mtb* infection who underwent therapeutic resection of infected tissue due to infection severity or treatment failure (*n* = 3). Patients with documented HIV coinfection were excluded. Postmortem autopsy specimens were acquired from the University of Texas Health Science Center (*n* = 3). Postmortem diagnosis of *Mtb* infection was confirmed with clinical history, culture, autopsy findings and IHC for *Mtb* antigens. All clinical details for specimens can be found in Extended Data Table 1. Serial sections (5 μm) of each specimen were stained with H&E and inspected by an anatomic pathologist to screen for the presence of granulomatous inflammation. Regions with histologically solid granulomas or cellular granulomatous inflammation surrounding central necrosis were included. Regions with excessive fibrosis or necrosis that were sparsely cellular or acellular were excluded. Two 500 μm × 500 μm FOVs were chosen from each tissue block for imaging. No statistical methods were used to predetermine sample sizes.

### Nontuberculous granulomas and controls tissues

Regions of granulomatous inflammation from FFPE sarcoidosis and foreign body reactions from Stanford Health Care were chosen by an anatomic pathologist; 0.5-mm cores were selected and constructed into a tissue microarray. A pulmonary *M. avium* case was acquired from Stanford Health Care through selection criteria of positive for acid-fast *Bacillus* staining and polymerase chain reaction positivity for *M. avium* complex. A 5-μm serial section of this specimen was stained with H&E and inspected by an anatomic pathologist to screen for the presence of granulomatous inflammation. Control tissues of FFPE tonsil, spleen and placenta were acquired from Stanford Health Care. Small regions of each tissue were selected and placed in a tissue microarray.

### Incorporation of additional specimens

It should be noted that the original study cohort comprised 20 FOVs across 10 patients, and 10 FOVs from 5 patients were added a later date. Any additional steps taken to process these specimens differently or incorporate them into the dataset are indicated in the relevant sections below.

### Antibody preparation

Antibodies were conjugated to isotopic metal reporters as described previously^18^. Following conjugation antibodies were diluted in Candor PBS Antibody Stabilization solution (Candor Bioscience). Antibodies were either stored at 4 °C or lyophilized in 100 mM D-( + )-Trehalose dehydrate (Sigma-Aldrich) with ultrapure distilled H_2_O for storage at −20 °C. Before staining, lyophilized antibodies were reconstituted in a buffer of Tris (Thermo Fisher Scientific), sodium azide (Sigma-Aldrich), ultrapure water (Thermo Fisher Scientific) and antibody stabilizer (Candor Bioscience) to a concentration of 0.05 mg ml^−1^. The antibodies, metal reporters and staining concentrations are listed in Extended Data Table 2. A limitation of this study is that we did not have an antibody for labeling bacteria due to the inherent difficulty of antibody-based detection of *Mtb* in FFPE tissue.

### Tissue staining

Tissues were sectioned (5 μm section thickness) from tissue blocks on gold and tantalum-sputtered microscope slides. Slides were baked at 70 °C overnight, followed by deparaffinization and rehydration with washes in xylene (3×), 100% ethanol (2×), 95% ethanol (2×), 80% ethanol (1×), 70% ethanol (1×) and ddH_2_O with a Leica ST4020 Linear Stainer (Leica Biosystems). Tissues next underwent antigen retrieval by submerging sides in 3-in-1 Target Retrieval Solution (pH 9, DAKO Agilent) and incubating at 97 °C for 40 min in a Lab Vision PT Module (Thermo Fisher Scientific). After cooling to room temperature, slides were washed in 1× PBS IHC Washer Buffer with Tween 20 (Cell Marque) with 0.1% (w/v) bovine serum albumin (Thermo Fisher). Next, all tissues underwent two rounds of blocking, the first to block endogenous biotin and avidin with an Avidin/Biotin Blocking Kit (BioLegend). Tissues were then washed with wash buffer and blocked for 1 h at room temperature with 1× TBS IHC Wash Buffer with Tween 20 with 3% (v/v) normal donkey serum (Sigma-Aldrich), 0.1% (v/v) cold fish skin gelatin (Sigma-Aldrich), 0.1% (v/v) Triton X-100, and 0.05% (v/v) Sodium Azide. The first antibody cocktail was prepared in 1x TBS IHC Wash Buffer with Tween 20 with 3% (v/v) normal donkey serum (Sigma-Aldrich) and filtered through a 0.1-μm centrifugal filter (Millipore) prior to incubation with tissue overnight at 4 °C in a humidity chamber. Following the overnight incubation, slides were washed twice for 5 min in wash buffer. The second day, an antibody cocktail was prepared as described and incubated with the tissues for 1 h at 4 °C in a humidity chamber. Following staining, slides were washed twice for 5 min in wash buffer and fixed in a solution of 2% glutaraldehyde (Electron Microscopy Sciences) solution in low-barium PBS for 5 min. Slides were washed in PBS (1×), 0.1 M Tris at pH 8.5 (3×) and ddH_2_O (2×) and then dehydrated by washing in 70% ethanol (1×), 80% ethanol (1×), 95% ethanol (2×) and 100% ethanol (2×). Slides were dried under vacuum prior to imaging.

### MIBI-TOF imaging

Imaging was performed using a MIBI-TOF instrument with a Hyperion ion source. Xe^+^ primary ions were used to sequentially sputter pixels for a given FOV. The following imaging parameters were used: acquisition setting, 80 kHz; field size, 500 μm x 500 μm (TB, *M. avium* and controls) or 450 μm × 450 μm (sarcoidosis) at 1,024 ×1,024 pixels; dwell time, 4 ms; median gun current on tissue, 1.45 nA Xe^+^; ion dose, 3.38 nAmp h per mm^2^ (500-μm^2^ FOVs) and 3.75 nAmp h per mm^2^ (450 μm^2^ FOVs).

For samples added to the cohort at a later time, the following imaging parameters were used: acquisition setting, 80 kHz; field size, 500 μm × 500 μm; dwell time, 1–2 ms; median gun current on tissue, 4.36 nA Xe^+^; ion dose, 2.54 × 5.08 nAmp h per mm^2^.

### Low-level image processing

Multiplexed image sets were extracted, slide background-subtracted, denoised and aggregate filtered as previously described^18^. All parameters for these steps can be found in Extended Data Table 2. In addition to these processing steps, image compensation was performed to account for signal spillover due to adducts and oxides for the following interferences: collagen-1 to IDO1 and Lag3, H3K9Ac to pan-CK and MPO, Chym/Tryp to MPO, Ki-67 to CD209, CD20 to CD16, CD16 to IFN-γ, CD11c to IDO1 and HLA-DR-DQ-DP to CD11b.

### Single-cell segmentation

Nuclear segmentation was performed using an adapted version of DeepCell^20^. DeepCell is a convolutional neural network that can be trained to predict single-cell segmentation across a range of biological platforms. One of the key challenges with segmentation of tissue data is the highly overlapping nature of adjacent nuclei. Previous work found that, although DeepCell is able to generate accurate segmentations using just a nuclear channel, it made errors where the tissue was very densely packed^18^. To improve the accuracy on these densely packed cells, especially immune cells, a modified version of the network was trained which included both a nuclear channel and a membrane channel as the basis for prediction.

First, training data were generating by manually annotating all of the nuclei in five separate images taken from a MIBI-TOF study of patients with melanoma, where each image was stained with HH3 to identify nuclei and Na^+^K^+^ATPase to identify the cell membrane (Extended Data Fig. 1d). For each image, the ImageJ platform was used to identify the location of each unique nuclei, which was then used to generate three distinct masks: one representing the interior of each nuclei, one representing the border of each nuclei and one representing the background of the image (which is all pixels not belonging to the first two classes). These three masks were created for each of the five images.

These masks, along with the image data, were used to train DeepCell. Each image was divided into overlapping crops of 64 × 64 pixels. Each crop was randomly flipped, rotated and sheared during training to further augment the available data. Stochastic gradient descent was used to train the network, with the performance assessed on a held-out portion of the data not seen by the network during training. This was combined with early stopping to prevent overfitting to the training data.

The network was trained to the predict which of the three classes each pixel in an image belongs to. The output of the network was a probability mask representing the confidence of the network in assigning each pixel to one of the three classes. All MIBI-TOF images from our cohort were provided as input to the network, with HH3 as the nuclear channel and CD45 as the membrane channel. To generate single-cell segments, the probability mask for the nuclear interior was thresholded, smoothed and run through the watershed algorithm as previously described^18^. Post-processing parameters such as the background threshold and smooth value were manually defined to balance the tradeoff between oversegmenting and undersegmenting the cells. Finally, we applied a three-pixel radial expansion around each nucleus to define the cell object boundaries.

A correction was applied to FOVs that contained MNGCs. Each MNGC was identified using a combination of HH3, CD45 and Vimentin and manually segmented in ImageJ to produce a mask of each MNGC. All pixels within the binary mask were reassigned to belong to the MNGC cell object(s). In total, 15 of 30 regions imaged contained MNGCs, with the number of MNGCs per field ranging from 1 to 9.

### Single-cell phenotyping and composition

Single-cell data were extracted for all cell objects and area normalized. Cells with a sum of <0.1 area-normalized counts across all lineage channels were excluded from analysis. Single-cell data were linearly scaled with a scaling factor of 100 and asinh-transformed with a cofactor of 5. All mass channels were scaled to the 99.9th percentile. In order to assign each cell to a lineage, the FlowSOM clustering algorithm was used in iterative rounds with the Bioconductor ‘FlowSOM’ package in R (ref. ^21^). The first clustering round separated cells into four major lineages using the ‘Metaclustering_consensus’ function (immune, epithelial, fibroblast and endothelial). Immune cells were then clustered again to delineate B cells, CD4^+^ T cells, CD8^+^ T cells, T_reg_ cells, neutrophils, mast cells and mononuclear phagocytes (macrophages, monocytes and DCs). Immune cells with an expression profile not consistent with any of those subsets were annotated as ‘other immune.’ Lastly, the mononuclear phagocytes were clustered to 25 metaclusters that were merged into 7 groups. Giant cells were manually identified. γδ T cells were annotated as T cells with CD3 signal greater than or equal to the mean expression of CD4^+^ T cells and TCR-δ signal >0.5 normalized expression. CD163 macrophages were identified as those myeloid cells with CD163 signal >0.5 normalized expression. Criteria used for assigning cell phenotypes can be found in Extended Data Table 3. Single-cell data that were collected for samples added to the cohort at a later date were extracted and normalized as described above, with 99.9th-percentile scaling done separately for the batch. In order to assign each cell to a lineage, the FlowSOM clustering algorithm was used in iterative rounds, and each resulting cluster was manually mapped to one of the original 20 phenotypic clusters. CD209^+^ DCs, CD14^+^CD16^+^ monocytes and epithelial cells were not annotated in samples 4–6 and 15 due to exclusion of CD209, CD16 and pan-Keratin in the panel used to stained these additional specimens. The relative abundance of all major lineages was calculated out of total cells per FOV, and the relative frequency of immune cell subsets was calculated out of total immune cells per FOV.

### Protein enrichment analysis

A spatial enrichment approached was used as previously described^18,74^ to identify patterns of protein enrichment or exclusion across all protein pairs in all samples. HH3, Na^+^K^+^ATPase and HLA class 1 were excluded from the analysis. For each pair of markers, X and Y, the number of times cells positive for protein X was within a ~50-μm radius of cells positive for protein Y was counted. Thresholds for positivity were customized to each marker individually using a silhouette scanning approach from the MetaCyto software in R (ref. ^75^). Thresholds were validated both by visual inspection of positive and negative cells in image sets and by inspection of the threshold overlaid on a histogram of signal distribution in single cells per marker (Extended Data Fig. 1e). A null distribution was produced by performing 1,000 bootstrap permutations where the locations of cells positive for protein X and Y were randomized. A z-score was calculated comparing the number of true cooccurrences of cells positive for protein X and Y relative to the null distribution. For each pair of proteins, X and Y, the average z-score was calculated across all TB FOVs. Next, all positive enrichments between protein pairs (average z-score >0, excluded self–self protein enrichment scores) were used to produce a weighted, undirected network, where the nodes are the individual markers and the edge weights are proportional to the average z-score (where a higher z-score indicates a shorter edge length). The leading eigenvector algorithm for community detection was used to identify protein enrichment communities in this network^76^.

### Spatial-LDA

Spatial-LDA is an adaptation of LDA, a machine-learning approach used to model topics in text documents, where topics consist of words with a high probability of co-occurrence, allowing mapping of topics to abstract definitions (e.g., [‘cat’, ‘frog’, ‘horse] → ‘animals’). Spatial-LDA builds on this paradigm by representing CPMs as documents and cell types as words. Spatial-LDA was conducted to identify topics (here referred to as MEs) across 26 TB FOVs. This included 20 FOVs from 10 patients in the current cohort along with 6 FOVs from 3 patients that were excluded from later analysis due to presence of HIV coinfection. All input data for producing the spatial-LDA model can be found in the data and code availability sections. Cell types with fewer than 100 cells across the entire cohort were excluded from analysis (γδ T cells and CD209^+^ DCs). Furthermore, MNGCs were excluded due to their cell size. Spatial-LDA was implemented as described previously^25^, with *d* = 1,000, a spatial radius *r* = 50 μm to complement the protein enrichment analysis and an ME number of *n* = 8. The ME number was determined empirically. For each FOV, a MaxPM was produced by classifying each cell to the ME with the highest probability and coloring that cell by its ME and probability. The 10 FOVs added at a later date were not included in generation of the spatial-LDA model but were assigned topic probabilities based on the same model built on the original 26 FOVs using the same cell types and spatial parameters as input. The cell type preferences for each ME were defined by assessing the mean probability for all cell types across all MEs. The mean expression for each functional marker across MEs was calculated by weighting protein expression by ME probability and calculating the mean of weighted expression values across markers and MEs. To cluster FOVs based on ME profile, the Pearson correlation coefficient was calculated between all pairs of TB FOVs based on the relative frequency of cells in each ME. The coefficients were used to hierarchically cluster the FOVs using complete linkage and a distance metric of 1 − correlation coefficient. To identify consensus clusters, the percent variance explained was measured across a range of 1–10 clusters. The elbow point of this plot was used to select the optimal number of clusters to capture the maximal variance in our dataset.

### Identification of the myeloid core

In order to assess which MEs represented the histologically defined myeloid core, binary masks of the myeloid core were generated for 15 FOVs. The masks were generated by first combining the signal of CD11c, CD11b, CD14, CD206, CD68 and PD-L1. The combined images were imported into ImageJ and hand-annotated using ROI annotation tools. The annotated ROI was exported as a binary mask. This mask was further processed in MATLAB to close any holes, exclude objects smaller than 1,000 pixels and dilate the mask to smooth edges. Next, cells were assigned to belonging to the myeloid core if they had complete overlap with the binary mask. Cells on the mask boundary or outside of the mask were designated as ‘nonmyeloid core.’ The proportion of cells in the myeloid core was assessed across each ME for the 15 FOVs and MEs with a median frequency in the myeloid core >50% were designated as myeloid core MEs.

### Myeloid cell UMAP visualization

UMAP embeddings were determined for all myeloid cells using the R implementation^77^ with the parameters n_neighbors = 15 and min_dist = 0.5 and the following markers: CD45, CD68, CD206, CD11c, CD11b, CD14, CD16, CD209, CD163, MPO/calprotectin and mast cell chymase/tryptase.

### Immunoregulatory protein analysis

Positivity thresholds for IDO1, PD-L1, PD-1 and Lag3 were automatically identified as described above. Immune control tissues tonsil, spleen and placenta were used to validate antibody performance. Correlation between IDO1 and PD-L1 was analyzed across the entire cohort in myeloid cells using Pearson correlation analysis. The frequencies of cells positive for IDO1 and PD-L1 were enumerated across all subsets. To assess PD-L1 and IDO1 positivity with respect to ME and cell subset, the total number of cells across all myeloid subsets per ME was pooled across all FOVs. The quantity of cells for each subset positive for IDO1 or PD-L1 was enumerated per ME. Any ME with <1% of the total for a subset was excluded from analysis. PD-1 and Lag3 expression was analyzed on lymphocytes or total immune cells. PD-1 and Lag3 expression was also analyzed on immune cells from a human TNBC cohort that was previously reported by our group^18^. Positivity for PD-1 and Lag3 for TNBC immune cells was defined as described in the original study (normalized signal >0.5).

### Regression analysis

In Fig. 1i and Extended Data Fig. 4h, linear regression was performed using the lm() command in R. The quality of the fit was evaluated and reported with the *r*^2^, Pearson *R*, and *P* value as measured by a two-tailed *t* test.

### ISH

ISH studies were performed with the commercially available RNAscope HD Reagent Kit (Advanced Cell Diagnostic) for single-plex chromogenic ISH. Following this protocol, FFPE tissue sections were baked, deparaffinized and incubated with RNAscope Hydrogen Peroxide solution for 10 min at room temperature. Antigen retrieval was carried out in 1× RNAscope Target retrieval solution for 30 min at 99 °C followed by protease digestion for 30 min at 40 °C in the HybEZ oven (Advanced Cell Diagnostic). All granuloma tissue along with human spleen and melanoma was probed for human *IFNG*, human *TGFB1*, positive control probe human *UBC* and negative control probe bacterial *DapB*. Additionally, commercially available HeLa cell pellet control slides were stained for *UBC* and *DapB*. Target probes were hybridized for 2 h at 40 °C using the HybEZ oven, followed by a series of six amplification steps. Following amplification, slides were stained using a chromogenic substrate (fast red) and counterstained using 50% Gill’s hematoxylin (American MasterTech) before evaluation by light microscopy, where each RNA transcript appeared as a distinct dot of red chromogen. QuPath software was used for quantification of results^78^. Briefly, each image was annotated to include only granulomatous inflammation and exclude surrounding tissue where present. Necrotic regions were also excluded. QuPath cell detection was used to detect cell objects in each image via the hematoxylin channel. Next subcellular detection was run on the fast-red channel with parameters set to include spot clusters. Total estimated spots, cell object counts and region areas were exported and analyzed in R. To separately analyze the myeloid core and lymphocytic zones of the granuloma, individual regions representing each histological zone were annotated based off the hematoxylin channel per image, and the signal was quantified as described previously.

### Cell composition analysis of sarcoidosis and TB

Single cells from sarcoidosis FOVs were segmented as described above. Single-cell data were extracted, transformed and normalized along with TB single-cell data. Single cells were included in the described FlowSOM clustering procedure.

### IHC of PD-L1 and IDO1

IHC for PD-L1 and IDO1 was performed using the antibody reagents listed in Extended Data Table 2 at a concentration of 1 μg ml^−1^. The IHC protocol mirrors the MIBI-TOF protocol, with the addition of blocking endogenous peroxidase activity with 3% H_2_O_2_ (Sigma-Aldrich) in ddH_2_O after epitope retrieval. On the second day of staining, instead of proceeding with the MIBI-TOF protocol, tissues were washed twice for 5 min in wash buffer and stained using ImmPRESS universal (Anti-Mouse/Anti-Rabbit) kit (Vector Laboratories).

### Whole-blood transcriptomic analysis

Publicly available gene expression datasets (Extended Data Table 4) were collected, annotated and used for meta-analysis conducted using MetaIntegrator^52^. Gene expression matrices were prepared for each dataset to determine effect sizes for genes of all proteins included in the MIBI-TOF analysis and an additional set of genes with similar biological function, such as *ICOS* and *CTLA4* (Extended Data Table 5). Summary effect sizes were calculated to assess gene expression differences across clinical groups (healthy, active TB, latent TB, end of treatment, TB progression and during treatment). For the catalysis treatment response cohort gene expression measurements at diagnosis of TB were correlated with matched total glycolytic activity index, a readout of PET-CT activity. A linear regression was fit between *CD274* gene expression and *TGAI* and the correlation was assessed with Pearson correlation analysis. To assess *CD274* and *PDCDLG2* gene expression overtreatment, expression values were normalized to the measurement taken at diagnosis (day 0). Gene expression data in the ACS were separated by progression status. Local regression was used to fit the gene expression data over time in each group. The significance of separation between progressors and non-progressors was calculated in two different time intervals using a Student’s t-test. We selected time points for this analysis by looking at the peak of separation (−1 to +1 months) and the earliest time point where we observed substantial separation between groups (i.e., where the error regions were not overlapping), which was around −6 months. The time interval was extended to capture a sufficient number of progressors around the −6-month time point to power the analysis. *CD274* expression was also compared in a ‘before/after’ analysis with the baseline measurement reflecting the earliest measurement prior to diagnosis and the diagnosis measurement representing the closest measurement taken to diagnosis with active TB (within 30 days before or after diagnosis). The predictive power of *CD274* expression was evaluated using ROC analysis. Negative and positive predicted values represent the proportion of samples that were actually negative/positive over the number of negative/positive samples reported by setting the threshold using Youden’s method.

### Statistics and reproducibility

All FOVs were included in all analyses. Data collection and analysis were not performed blind to the type of specimen. Processed images were displayed to show representative imaging data, but quantification of results was performed on unaltered images. For significance testing of cell, phenotypic and ME data, we conservatively applied tests that did not assume normality. *t* tests were applied for linear regression and correlation analyses in line with the assumption that errors and residuals are normally distributed. Statistical analysis of the transcriptomic data employed multiple hypothesis correction and significance testing as previously established^57^.

### Software

Image processing was conducted with MATLAB 2016a and MATLAB 2019b. Statistical analysis was conducted in MATLAB 2016a, MATLAB 2019b and R v3.6.2. Python 3.6 was used for implementation of spatial-LDA. Data visualization and plots were generated in R. Representative images were processed in Adobe Photoshop, and figures were prepared in Adobe Illustrator. Schematic visualizations were produced at https://biorender.com.

### Reporting Summary

Further information on research design is available in the Nature Research Reporting Summary linked to this article.

## Online content

Any methods, additional references, Nature Research reporting summaries, source data, extended data, supplementary information, acknowledgements, peer review information; details of author contributions and competing interests; and statements of data and code availability are available at 10.1038/s41590-021-01121-x.

### Supplementary information

Supplementary Information Extended Data Tables 1–5.

Reporting Summary

## Figures and Tables

**Extended Data Table 1:**
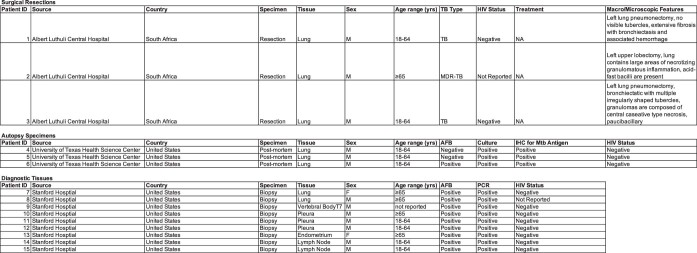
Tuberculosis granuloma cohort clinical information

**Extended Data Table 2:**
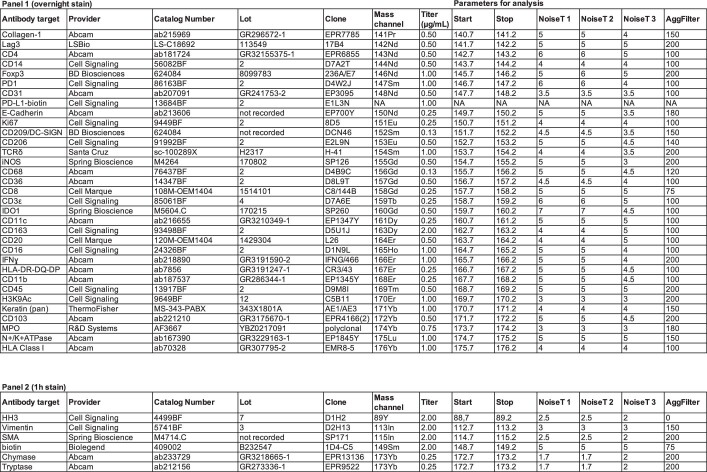
Multiplexed imaging antibody panel staining conditions and low-level processing parameters

**Extended Data Table 3:**
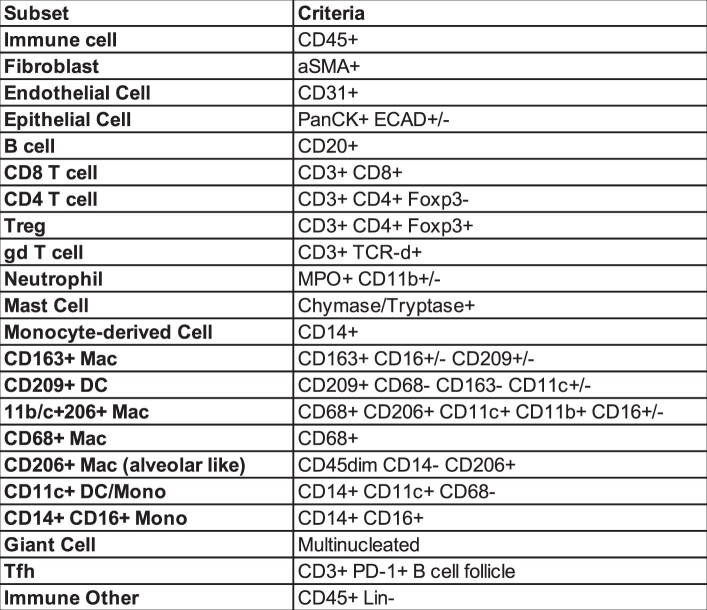
Cell phenotyping criteria

**Extended Data Table 4:**
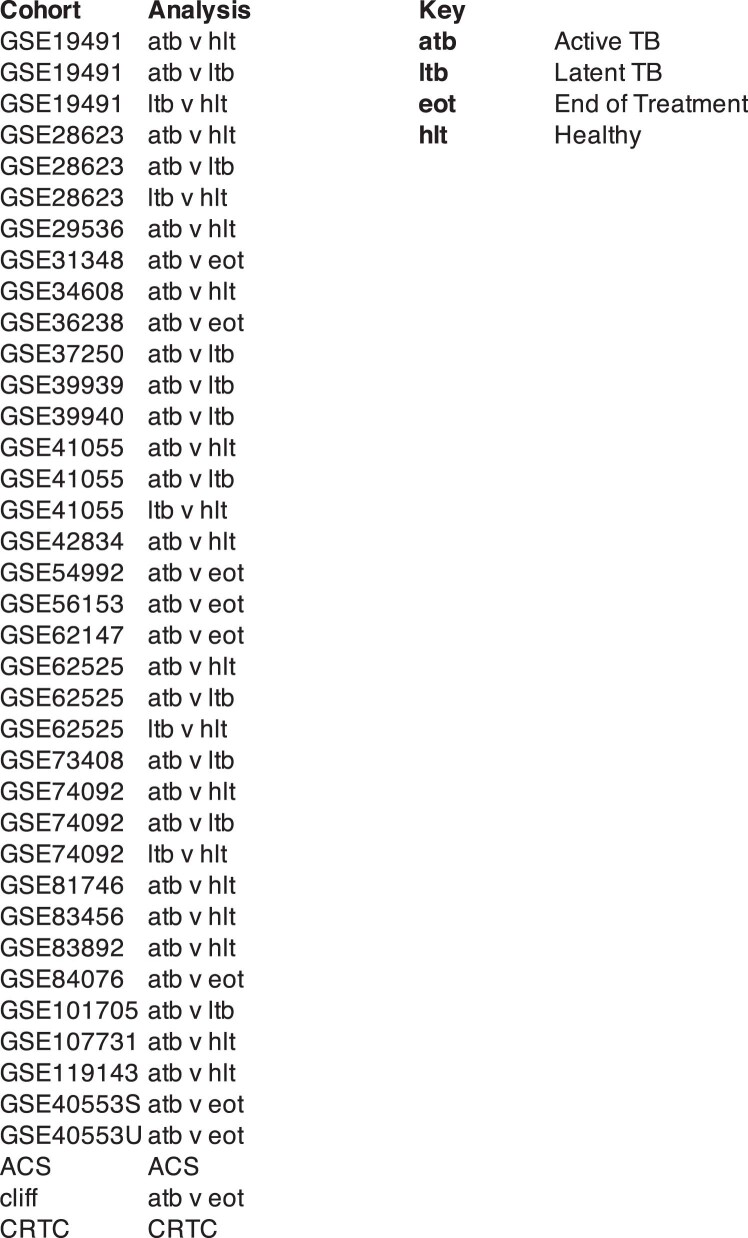
Gene Expression cohort description

**Extended Data Table 5:**
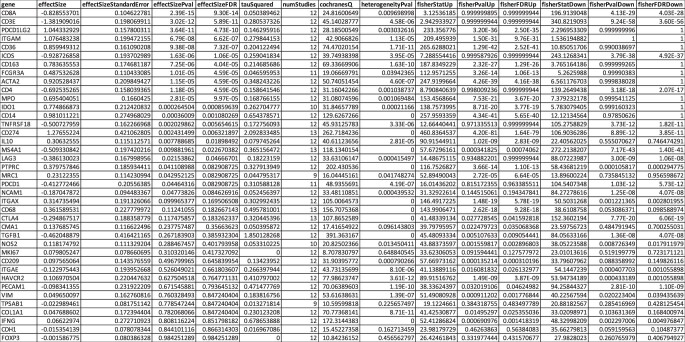
MetaIntegrator analysis of active TB and healthy controls

## Data Availability

All images and annotated single-cell data are deposited in Mendeley’s data repository and can be accessed using the following link: 10.17632/dr5fkgtrb6.

## Code availability

All custom code used to analyze data has been deposited in GitHub and can be accessed using the following link: https://github.com/angelolab/publications/tree/master/2022-McCaffrey_etal_HumanTB.
