# Author Correction: The immunoregulatory landscape of human tuberculosis granulomas

**DOI:** 10.1038/s41590-022-01178-2

**Published:** 2022-03-11

**Authors:** Erin F. McCaffrey, Michele Donato, Leeat Keren, Zhenghao Chen, Alea Delmastro, Megan B. Fitzpatrick, Sanjana Gupta, Noah F. Greenwald, Alex Baranski, William Graf, Rashmi Kumar, Marc Bosse, Christine Camacho Fullaway, Pratista K. Ramdial, Erna Forgó, Vladimir Jojic, David Van Valen, Smriti Mehra, Shabaana A. Khader, Sean C. Bendall, Matt van de Rijn, Daniel Kalman, Deepak Kaushal, Robert L. Hunter, Niaz Banaei, Adrie J. C. Steyn, Purvesh Khatri, Michael Angelo

**Affiliations:** 1 Department of Pathology, Stanford University School of Medicine, Stanford, CA USA; 2 Department of Medicine, Division of Biomedical Informatics Research, Stanford University School of Medicine, Stanford, CA USA; 3 Institute for Immunity, Transplantation and Infection, Stanford University School of Medicine, Stanford, CA USA; 4 Department of Molecular Cell Biology, Weizmann Institute of Science, Rehovot, Israel; 5 Calico Life Sciences LLC, South San Francisco, CA USA; 6 Department of Pathology, University of Wisconsin, Madison, WI USA; 7 Division of Biology and Bioengineering, California Institute of Technology, Pasadena, CA USA; 8 Africa Health Research Institute, University of KwaZulu-Natal, Durban, South Africa; 9 Texas Biomedical Research Institute, San Antonio, TX USA; 10 Department of Molecular Microbiology, Washington University School of Medicine, St. Louis, MO USA; 11 Department of Pathology and Laboratory Medicine, Emory University School of Medicine, Atlanta, GA USA; 12 Southwest National Primate Research Center, Texas Biomedical Research Institute, San Antonio, TX USA; 13 Department of Pathology and Laboratory Medicine, University of Texas Health Sciences Center at Houston, Houston, TX USA; 14 Division of Infectious Diseases & Geographic Medicine, Department of Medicine, Stanford University, Stanford, CA USA; 15 Department of Microbiology, University of Alabama at Birmingham, Birmingham, AL USA

**Keywords:** Tuberculosis, Imaging the immune system, Infection

Correction to: *Nature Immunology* 10.1038/s41590-021-01121-x, published online 20 January 2022.

In the version of the article originally published, the link provided in the Code availability section was invalid and has now been replaced in the HTML and PDF versions of the article with the following: https://github.com/angelolab/publications/tree/master/2022-McCaffrey_etal_HumanTB.

