# Supplementary material for: The immunoregulatory landscape of human tuberculosis granulomas
Source: Nat Immunol. 2022 Jan 20;23(2):318–29. doi: 10.1038/s41590-021-01121-x (PMC8810384; doi:10.1038/s41590-021-01121-x)
Supplement: Supplementary file 1 — Extended Data Tables 1–5. [file 41590_2021_1121_MOESM1_ESM.pdf]

nature portfolio

https://doi.org/10.1038/s41590-021-01121-x

Supplementary information

The immunoregulatory landscape of human tuberculosis granulomas

In the format provided by the authors and unedited

Extended Data Table 1. Tuberculosis granuloma cohort clinical information

Surgical Resections

Patient ID	Source	Country	Specimen	Tissue	Sex	Age range (yrs)	TB Type	HIV Status	Treatment	Macro/Microscopic Features
1	Albert Luthuli Central Hospital	South Africa	Resection	Lung	M	18-64	TB	Negative	NA	Left lung pneumonectomy, no visible tubercles, extensive fibrosis with bronchiectasis and associated hemorrhage
2	Albert Luthuli Central Hospital	South Africa	Resection	Lung	M	≥65	MDR-TB	Not Reported	NA	Left upper lobectomy, lung contains large areas of necrotizing granulomatous inflammation, acid-fast bacilli are present
3	Albert Luthuli Central Hospital	South Africa	Resection	Lung	M	18-64	TB	Negative	NA	Left lung pneumonectomy, bronchiectatic with multiple irregularly shaped tubercles, granulomas are composed of central caseative type necrosis, paucibacillary

Autopsy Specimens

Patient ID	Source	Country	Specimen	Tissue	Sex	Age range (yrs)	AFB	Culture	IHC for Mtb Antigen	HIV Status
4	University of Texas Health Science Center	United States	Post-mortem	Lung	M	18-64	Negative	Positive	Positive	Negative
5	University of Texas Health Science Center	United States	Post-mortem	Lung	M	18-64	Negative	Positive	Positive	Negative
6	University of Texas Health Science Center	United States	Post-mortem	Lung	M	18-64	Positive	Positive	Positive	Negative

Diagnostic Tissues

Patient ID	Source	Country	Specimen	Tissue	Sex	Age range (yrs)	AFB	PCR	HIV Status
7	Stanford Hosptial	United States	Biopsy	Lung	F	≥65	Positive	Positive	Negative
8	Stanford Hosptial	United States	Biopsy	Lung	M	≥65	Positive	Positive	Not Reported
9	Stanford Hosptial	United States	Biopsy	Vertebral BodyT7	M	not reported	Positive	Positive	Negative
10	Stanford Hosptial	United States	Biopsy	Pleura	M	≥65	Positive	Positive	Negative
11	Stanford Hosptial	United States	Biopsy	Pleura	M	18-64	Positive	Positive	Negative
12	Stanford Hosptial	United States	Biopsy	Pleura	M	18-64	Positive	Positive	Negative
13	Stanford Hosptial	United States	Biopsy	Endometrium	F	≥65	Positive	Positive	Negative
14	Stanford Hosptial	United States	Biopsy	Lymph Node	M	18-64	Positive	Positive	Negative
15	Stanford Hosptial	United States	Biopsy	Lymph Node	M	18-64	Positive	Positive	Negative

Extended Data Table 2. Multiplexed imaging antibody panel staining conditions and low-level processing parameters

Panel 1 (overnight stain)

							Parameters for analysis					
**Antibody target**	**Provider**	**Catalog Number**	**Lot**	**Clone**	**Mass channel**	**Titer (µg/mL)**	**Start**	**Stop**	**NoiseT 1**	**NoiseT 2**	**NoiseT 3**	**AggFilter**
Collagen-1	Abcam	ab215969	GR296572-1	EPR7785	141Pr	0.50	140.7	141.2	5	5	4	150
Lag3	LSBio	LS-C18692	113549	17B4	142Nd	0.50	141.7	142.2	5	5	5	200
CD4	Abcam	ab181724	GR32155375-1	EPR6855	143Nd	0.50	142.7	143.2	6	6	5	100
CD14	Cell Signaling	56082BF	2	D7A2T	144Nd	0.50	143.7	144.2	4	4	4	100
Foxp3	BD Biosciences	624084	8099783	236A/E7	146Nd	1.00	145.7	146.2	5	6	5	200
PD1	Cell Signaling	86163BF	2	D4W2J	147Sm	1.00	146.7	147.2	6	6	4	100
CD31	Abcam	ab207091	GR241753-2	EP3095	148Nd	0.50	147.7	148.2	3.5	3.5	3.5	100
PD-L1-biotin	Cell Signaling	13684BF	2	E1L3N	NA	1.00	NA	NA	NA	NA	NA	NA
E-Cadherin	Abcam	ab213606	not recorded	EP700Y	150Nd	0.25	149.7	150.2	5	5	3.5	180
Ki67	Cell Signaling	9449BF	2	8D5	151Eu	0.25	150.7	151.2	4	4	4	100
CD209/DC-SIGN	BD Biosciences	624084	not recorded	DCN46	152Sm	0.13	151.7	152.2	4.5	4.5	3.5	150
CD206	Cell Signaling	91992BF	2	E2L9N	153Eu	0.50	152.7	153.2	5	5	4.5	140
TCRδ	Santa Cruz	sc-100289X	H2317	H-41	154Sm	1.00	153.7	154.2	4	4	3.5	200
iNOS	Spring Bioscience	M4264	170802	SP126	155Gd	0.50	154.7	155.2	5	5	3	200
CD68	Abcam	76437BF	2	D4B9C	156Gd	0.13	155.7	156.2	5	5	4.5	120
CD36	Abcam	14347BF	2	D8L9T	157Gd	0.50	156.7	157.2	4.5	4.5	4	100
CD8	Cell Marque	108M-OEM1404	1514101	C8/144B	158Gd	0.25	157.7	158.2	5	5	5	75
CD3ε	Cell Signaling	85061BF	4	D7A6E	159Tb	0.25	158.7	159.2	6	6	5	100
IDO1	Spring Bioscience	M5604.C	170215	SP260	160Gd	0.50	159.7	160.2	7	7	4.5	100
CD11c	Abcam	ab216655	GR3210349-1	EP1347Y	161Dy	0.25	160.7	161.2	5	5	5	100
CD163	Cell Signaling	93498BF	2	D5U1J	163Dy	2.00	162.7	163.2	4	4	5	100
CD20	Cell Marque	120M-OEM1404	1429304	L26	164Er	0.50	163.7	164.2	4	4	5	100
CD16	Cell Signaling	24326BF	2	D1N9L	165Ho	1.00	164.7	165.2	5	5	4	100
IFNγ	Abcam	ab218890	GR3191590-2	IFNG/466	166Er	1.00	165.7	166.2	5	5	4	200
HLA-DR-DQ-DP	Abcam	ab7856	GR3191247-1	CR3/43	167Er	0.25	166.7	167.2	5	5	4.5	100
CD11b	Abcam	ab187537	GR286344-1	EP1345Y	168Er	0.25	167.7	168.2	5	5	4.5	100
CD45	Cell Signaling	13917BF	2	D9M8I	169Tm	0.50	168.7	169.2	5	5	5	200
H3K9Ac	Cell Signaling	9649BF	12	C5B11	170Er	1.00	169.7	170.2	3	3	3	200
Keratin (pan)	ThermoFisher	MS-343-PABX	343X1801A	AE1/AE3	171Yb	1.00	170.7	171.2	4	4	4	150
CD103	Abcam	ab221210	GR3175670-1	EPR4166(2)	172Yb	0.50	171.7	172.2	5	5	4.5	200
MPO	R&D Systems	AF3667	YBZ0217091	polyclonal	174Yb	0.75	173.7	174.2	3	3	3	180
N+/K+ATPase	Abcam	ab167390	GR3229163-1	EP1845Y	175Lu	1.00	174.7	175.2	5	5	5	150
HLA Class I	Abcam	ab70328	GR307795-2	EMR8-5	176Yb	1.00	175.7	176.2	4	4	4	100

Panel 2 (1h stain)

**Antibody target**	**Provider**	**Catalog Number**	**Lot**	**Clone**	**Mass channel**	**Titer**	**Start**	**Stop**	**NoiseT 1**	**NoiseT 2**	**NoiseT 3**	**AggFilter**
HH3	Cell Signaling	4499BF	7	D1H2	89Y	2.00	88,7	89.2	2.5	2.5	2	0
Vimentin	Cell Signaling	5741BF	3	D2H13	113In	2.00	112.7	113.2	3	3	3	150
SMA	Spring Bioscience	M4714.C	not recorded	SP171	115In	2.00	114.7	115.2	2.5	2.5	2	200
biotin	Biolegend	409002	B232547	1D4-C5	149Sm	2.00	148.7	149.2	5	5	5	75
Chymase	Abcam	ab233729	GR3218665-1	EPR13136	173Yb	0.25	172.7	173.2	1.7	1.7	2	200
Tryptase	Abcam	ab212156	GR273336-1	EPR9522	173Yb	0.25	172.7	173.2	1.7	1.7	2	200

Extended Data Table 3. Cell phenotyping criteria

Subset	Criteria
**Immune cell**	CD45+
**Fibroblast**	aSMA+
**Endothelial Cell**	CD31+
**Epithelial Cell**	PanCK+ ECAD+/-
**B cell**	CD20+
**CD8 T cell**	CD3+ CD8+
**CD4 T cell**	CD3+ CD4+ Foxp3-
**Treg**	CD3+ CD4+ Foxp3+
**gd T cell**	CD3+ TCR-d+
**Neutrophil**	MPO+ CD11b+/-
**Mast Cell**	Chymase/Tryptase+
**Monocyte-derived Cell**	CD14+
**CD163+ Mac**	CD163+ CD16+/- CD209+/-
**CD209+ DC**	CD209+ CD68- CD163- CD11c+/-
**11b/c+206+ Mac**	CD68+ CD206+ CD11c+ CD11b+ CD16+/-
**CD68+ Mac**	CD68+
**CD206+ Mac (alveolar like)**	CD45dim CD14- CD206+
**CD11c+ DC/Mono**	CD14+ CD11c+ CD68-
**CD14+ CD16+ Mono**	CD14+ CD16+
**Giant Cell**	Multinucleated
**Tfh**	CD3+ PD-1+ B cell follicle
**Immune Other**	CD45+ Lin-

Extended Data Table 4. Gene expression cohort description

Cohort	Analysis
GSE19491	atb v hlt
GSE19491	atb v ltb
GSE19491	ltb v hlt
GSE28623	atb v hlt
GSE28623	atb v ltb
GSE28623	ltb v hlt
GSE29536	atb v hlt
GSE31348	atb v eot
GSE34608	atb v hlt
GSE36238	atb v eot
GSE37250	atb v ltb
GSE39939	atb v ltb
GSE39940	atb v ltb
GSE41055	atb v hlt
GSE41055	atb v ltb
GSE41055	ltb v hlt
GSE42834	atb v hlt
GSE54992	atb v eot
GSE56153	atb v eot
GSE62147	atb v eot
GSE62525	atb v hlt
GSE62525	atb v ltb
GSE62525	ltb v hlt
GSE73408	atb v ltb
GSE74092	atb v hlt
GSE74092	atb v ltb
GSE74092	ltb v hlt
GSE81746	atb v hlt
GSE83456	atb v hlt
GSE83892	atb v hlt
GSE84076	atb v eot
GSE101705	atb v ltb
GSE107731	atb v hlt
GSE119143	atb v hlt
GSE40553S	atb v eot
GSE40553U	atb v eot
ACS	ACS
cliff	atb v eot
CRTC	CRTC

Key	
**atb**	Active TB
**ltb**	Latent TB
**eot**	End of Treatment
**hlt**	Healthy

Extended Data Table 5. MetaIntegrator analysis of active TB and healthy controls.

gene	effectSize	effectSizeStandardError	effectSizePval	effectSizeFDR	tauSquared	numStudies	cochranesQ	heterogeneityPval	fisherStatUp	fisherPvalUp	fisherFDRUp	fisherStatDown	fisherPvalDown	fisherFDRDown
CD8A	-0.828553701	0.104622781	2.39E-15	9.30E-14	0.050389462	12	24.81600649	0.009698998	3.12536185	0.999999895	0.999999944	196.9139048	4.13E-29	4.03E-28
CD3E	-1.381909016	0.198069911	3.02E-12	5.89E-11	0.280537326	12	45.14028777	4.58E-06	2.942933927	0.999999944	0.999999944	340.8219093	9.24E-58	3.60E-56
PDCD1LG2	1.044332929	0.157800311	3.64E-11	4.73E-10	0.146295916	12	28.18500549	0.003032616	233.356776	3.20E-36	2.50E-35	2.296953309	0.999999996	1
ITGAM	1.076483238	0.199472155	6.79E-08	6.62E-07	0.279844153	12	42.9066826	1.13E-05	209.495939	1.50E-31	9.76E-31	1.536194882	1	1
CD36	0.859949312	0.161090208	9.38E-08	7.32E-07	0.204122494	12	74.47020154	1.71E-11	265.6288021	1.29E-42	2.52E-41	10.85051706	0.990038697	1
ICOS	-0.928726858	0.193702989	1.63E-06	1.06E-05	0.259041834	12	39.74938398	3.95E-05	7.288554416	0.999587926	0.999999944	243.1268341	3.79E-38	4.92E-37
CD163	0.783635553	0.174681187	7.25E-06	4.04E-05	0.214685686	12	69.33669906	1.63E-10	187.8349229	2.32E-27	1.29E-26	3.765164186	0.999999265	1
FCGR3A	0.487532628	0.110433085	1.01E-05	4.59E-05	0.046595953	11	19.06669791	0.03942365	112.9571255	3.26E-14	1.06E-13	5.2625988	0.99990383	1
ACTA2	0.920528437	0.209849427	1.15E-05	4.59E-05	0.348243226	12	50.74051454	4.60E-07	247.9199664	4.26E-39	4.16E-38	6.561176703	0.999838028	1
CD4	-0.692535265	0.158039365	1.18E-05	4.59E-05	0.158641546	12	31.16042266	0.001038737	8.790840639	0.998009236	0.999999944	139.2649438	3.18E-18	2.07E-17
MPO	0.695404051	0.1660425	2.81E-05	9.97E-05	0.168766155	12	31.08074596	0.001069484	153.4568664	7.53E-21	3.67E-20	7.379232178	0.999541125	1
IDO1	0.774866873	0.212420832	0.000264504	0.000859639	0.262704777	10	31.84657789	0.00021166	138.7573995	8.71E-20	3.77E-19	5.783079405	0.999160323	1
CD14	0.981011221	0.274968029	0.00036009	0.001080269	0.654378571	12	129.6267266	0	257.9593359	4.34E-41	5.65E-40	12.12134564	0.97850626	1
TNFRSF18	-0.500727959	0.162266968	0.002029862	0.005654615	0.172756093	12	45.93125783	3.33E-06	12.66440441	0.971335513	0.999999944	105.2758829	3.73E-12	1.82E-11
CD274	1.27655224	0.421062805	0.002431499	0.006321897	2.092833485	13	262.7184236	0	460.8364537	4.20E-81	1.64E-79	106.9036286	8.89E-12	3.85E-11
IL10	0.30632555	0.115112571	0.007788685	0.01898492	0.079745264	12	40.61123656	2.81E-05	90.91544911	1.02E-09	2.83E-09	22.40562025	0.555070627	0.746474291
MS4A1	-0.509330842	0.197420216	0.009881961	0.022670382	0.365156472	13	118.1340154	0	57.67296161	0.000341825	0.00074062	272.2138207	7.17E-43	1.40E-41
LAG3	-0.386130023	0.167998956	0.02153862	0.04666701	0.18223159	12	33.63106147	0.000415497	14.48675115	0.934882201	0.999999944	88.07223987	3.00E-09	1.06E-08
PTPRC	0.379757846	0.185934411	0.041108988	0.082908725	0.327913949	12	202.430536	0	116.7526827	3.66E-14	1.10E-13	58.43681219	0.000105817	0.000294775
MRC1	0.23122355	0.114230994	0.042952125	0.082908725	0.044795317	9	16.04445161	0.041748274	52.89490043	2.72E-05	6.64E-05	13.89600224	0.735845132	0.956598672
PDCD1	-0.412772466	0.20556385	0.04464316	0.082908725	0.310588128	11	48.9355691	4.19E-07	16.01436202	0.815172355	0.963385511	104.5407348	1.03E-12	5.73E-12
NCAM1	-0.187047872	0.094483367	0.04773826	0.084626916	0.052456397	12	33.48110851	0.000439532	31.32922614	0.144515061	0.194347841	84.27278616	1.25E-08	4.07E-08
ITGAX	0.314735494	0.191326066	0.099965377	0.169506508	0.302992435	12	105.0064573	0	146.4917225	1.48E-19	5.78E-19	50.5031268	0.001221365	0.002801955
CD68	0.361589531	0.227779972	0.11241055	0.182667143	0.495781001	13	156.7075368	0	143.9906471	2.62E-18	9.28E-18	38.6108758	0.053086371	0.098588974
CTLA4	-0.294867517	0.188358779	0.117475857	0.183262337	0.320445396	13	107.8652589	0	41.48339134	0.027728545	0.041592818	152.3602194	7.77E-20	6.06E-19
CMA1	0.137685745	0.116622496	0.237757487	0.35663623	0.050395872	12	17.41654922	0.096143803	39.79795957	0.022479723	0.035068368	23.59756723	0.484791945	0.700255031
TGFB1	-0.462048879	0.416421165	0.267183903	0.385932304	1.850128268	12	391.363167	0	45.48093334	0.005107633	0.009054441	84.05633166	1.36E-08	4.07E-08
NOS2	0.118174792	0.111329204	0.288467457	0.401793958	0.053310225	10	20.82502366	0.013450411	43.88373597	0.001559817	0.002896803	38.05223588	0.008726349	0.017911979
MKI67	0.079805247	0.078660695	0.310320146	0.417327092	0	12	8.707830797	0.648840545	33.62306053	0.091594441	0.127577972	23.01013616	0.519199779	0.723171121
CD209	0.097565064	0.143576559	0.496799965	0.645839954	0.13423952	12	31.90395772	0.000790266	57.66973162	0.000135214	0.000310196	33.79607962	0.088358992	0.149826116
ITGAE	-0.122975443	0.193952668	0.526049021	0.661803607	0.266397944	12	43.73135699	8.10E-06	41.13889116	0.016081832	0.026132977	54.1447239	0.000407703	0.001055898
HAVCR2	0.106970504	0.220447632	0.627504518	0.764771131	0.410797002	12	77.98623747	3.61E-12	89.91516762	1.49E-09	3.87E-09	53.94734189	0.000433189	0.001055898
PECAM1	-0.098341355	0.231922209	0.671545881	0.793645132	0.471477769	12	70.06389603	1.19E-10	38.33624397	0.032019106	0.04624982	94.25844327	2.81E-10	1.10E-09
VIM	0.049650097	0.162760816	0.760328493	0.847240404	0.183816756	12	53.61638631	1.39E-07	51.49080928	0.000911202	0.001776844	40.22567594	0.020223404	0.039435639
TPSAB1	-0.022989461	0.081751142	0.778547244	0.847240404	0.013271814	9	10.59599818	0.225657497	19.1224661	0.384318755	0.483497789	20.88182567	0.285416969	0.428125454
COL1A1	0.047688602	0.172394404	0.782068066	0.847240404	0.230123208	12	70.77368141	8.71E-11	41.42530877	0.01495297	0.025355036	33.02098971	0.103631369	0.168400974
IFNG	0.06622974	0.272710923	0.808116224	0.851798182	0.678653888	12	172.3144383	0	52.41286824	0.000690976	0.001418319	48.32998209	0.002297006	0.004976847
CDH1	-0.015354139	0.078078344	0.844101116	0.866314303	0.016967086	12	15.45227358	0.162713459	23.98179729	0.46263863	0.56384083	35.66279813	0.059159563	0.10487377
FOXP3	-0.001586775	0.080386328	0.984251289	0.984251289	0	12	10.84236152	0.456562797	26.42461843	0.331977444	0.431570677	27.9828023	0.260765979	0.406794927
