# Supplementary material for: The immunoregulatory landscape of human tuberculosis granulomas
Source: Nat Immunol. 2022 Jan 20;23(2):318–29. doi: 10.1038/s41590-021-01121-x (PMC8810384; doi:10.1038/s41590-021-01121-x)
Supplement: Supplementary file 2 — Reporting Summary [file 41590_2021_1121_MOESM2_ESM.pdf]

nature research

Corresponding author(s): Michael Angelo

Last updated by author(s): Dec 10, 2021

Reporting Summary

Nature Research wishes to improve the reproducibility of the work that we publish. This form provides structure for consistency and transparency in reporting. For further information on Nature Research policies, see our Editorial Policies and the Editorial Policy Checklist.

Statistics

For all statistical analyses, confirm that the following items are present in the figure legend, table legend, main text, or Methods section.

n/a	Confirmed	
☐	☒	The exact sample size (*n*) for each experimental group/condition, given as a discrete number and unit of measurement
☐	☒	A statement on whether measurements were taken from distinct samples or whether the same sample was measured repeatedly
☐	☒	The statistical test(s) used AND whether they are one- or two-sided<br>*Only common tests should be described solely by name; describe more complex techniques in the Methods section.*
☐	☒	A description of all covariates tested
☐	☒	A description of any assumptions or corrections, such as tests of normality and adjustment for multiple comparisons
☐	☒	A full description of the statistical parameters including central tendency (e.g. means) or other basic estimates (e.g. regression coefficient) AND variation (e.g. standard deviation) or associated estimates of uncertainty (e.g. confidence intervals)
☐	☒	For null hypothesis testing, the test statistic (e.g. *F*, *t*, *r*) with confidence intervals, effect sizes, degrees of freedom and *P* value noted<br>*Give P values as exact values whenever suitable.*
☒	☐	For Bayesian analysis, information on the choice of priors and Markov chain Monte Carlo settings
☐	☒	For hierarchical and complex designs, identification of the appropriate level for tests and full reporting of outcomes
☐	☒	Estimates of effect sizes (e.g. Cohen's *d*, Pearson's *r*), indicating how they were calculated

Our web collection on statistics for biologists contains articles on many of the points above.

Software and code

Policy information about availability of computer code

Data collection	MIBI-TOF data collection software (MiniSIMS version 5.5.4.0)
Data analysis	Matlab 2016a, Matlab 2019b, R 3.6.2 (all packages are listed in a document at the Github link listed below), Python3.6, QuPath 0.2.3. All custom code available at: https://github.com/angelolab/publications/2022-McCaffrey_etal_HumanTB

For manuscripts utilizing custom algorithms or software that are central to the research but not yet described in published literature, software must be made available to editors and reviewers. We strongly encourage code deposition in a community repository (e.g. GitHub). See the Nature Research guidelines for submitting code & software for further information.

Data

Policy information about availability of data

All manuscripts must include a data availability statement. This statement should provide the following information, where applicable:

- Accession codes, unique identifiers, or web links for publicly available datasets
- A list of figures that have associated raw data
- A description of any restrictions on data availability

All images and annotated single cell data can be accessed through Mendeley's data repository at the following link: DOI: 10.17632/dr5fkgtrb6. The GEO accession numbers for all transcriptomic data used in our meta-analysis can be found in Extended Data Table 4.

Field-specific reporting

Please select the one below that is the best fit for your research. If you are not sure, read the appropriate sections before making your selection.

☒ Life sciences ☐ Behavioural & social sciences ☐ Ecological, evolutionary & environmental sciences

For a reference copy of the document with all sections, see nature.com/documents/nr-reporting-summary-flat.pdf

Life sciences study design

All studies must disclose on these points even when the disclosure is negative.

Sample size	Sample size was not statistically determined. Cohort sample size was based on specimen availability and histological selection criteria to screen samples available from Stanford, AHRI, and University of Texas Health Science Center tissue repositories.
Data exclusions	No data excluded
Replication	All cohort specimens were prepared and analyzed alongside control tissue specimens to validate the technical performance of all reagents, instrument settings, and analysis parameters used in the study. Antibody reagents were extensively validated on control tissues with immunohistochemistry and MIBI-TOF analysis. Data collection was performed on all specimens once.
Randomization	This is not relevant because there were no separate experimental groups in this study. The cohort was a retrospective analysis of archival clinical tissue specimens. Controls were used for technical validation of antibodies, but not for comparative analysis. All samples were prepared together and imaged in a random order.
Blinding	The study did not require blinding as it was a retrospective analysis of archival clinical tissue specimens. That said, data collection and analysis were not performed blind to the type of specimen.

Reporting for specific materials, systems and methods

We require information from authors about some types of materials, experimental systems and methods used in many studies. Here, indicate whether each material, system or method listed is relevant to your study. If you are not sure if a list item applies to your research, read the appropriate section before selecting a response.

Materials & experimental systems

n/a	Involved in the study	
☐	☒	Antibodies
☐	☒	Eukaryotic cell lines
☒	☐	Palaeontology and archaeology
☒	☐	Animals and other organisms
☐	☒	Human research participants
☒	☐	Clinical data
☒	☐	Dual use research of concern

Methods

n/a	Involved in the study	
☒	☐	ChIP-seq
☒	☐	Flow cytometry
☒	☐	MRI-based neuroimaging

Antibodies

Antibodies used	Study used 39 primary antibodies. See extended data table 2 for all requested information of each reagent including the precise working concentration used (called "Titer").
Validation	All reagents were validated in-house with chromogenic immunohistochemistry on FFPE human control tissues. Following this stage of validation all antibodies were metal-labeled and further tested with MIBI-TOF. Imaging data for all antibodies in control tissues (spleen, tonsil, placenta) can be found in our data repository.

Eukaryotic cell lines

Policy information about cell lines

Cell line source(s)	Commercial control slides containing FFPE cultured cell pellets of human HeLa cells from Advanced Cell Diagnostic (ACD)
Authentication	NA
Mycoplasma contamination	NA

April 2020

Commonly misidentified lines (See ICLAC register)	NA

Human research participants

Policy information about studies involving human research participants

Population characteristics	Study did not directly involve human participants, but used archival clinical specimens from individuals with active Mycobacterium tuberculosis infection. Individuals were treated in the U.S. (n=12) or South Africa (n=3). Study included both male and female individuals with reported ages of 19-88 years.
Recruitment	Study was done on archival clinical specimens from tissue repositories. No recruitment was done.
Ethics oversight	All human samples were acquired in accordance with Institutional Review Board (IRB) protocol #46586: “Generation of an Immune Atlas of Human Tuberculosis Granulomas with Multiplexed Ion Beam Imaging.”

Note that full information on the approval of the study protocol must also be provided in the manuscript.
